# Dynamics and Eco‐Genomics of Baltic Sea Nitrifiers: Seasonality, Niches, Interactions and Genomic Uniqueness

**DOI:** 10.1111/1462-2920.70215

**Published:** 2026-01-07

**Authors:** Sukki Sookyoung Kim, Elisa D'Agostino, David M. Needham

**Affiliations:** ^1^ Ocean Ecosystems Biology Unit, Marine Ecology Division, GEOMAR Helmholtz Centre for Ocean Research Kiel Kiel Germany; ^2^ Faculty of Mathematics and Natural Sciences Kiel University Kiel Germany

## Abstract

Nitrification is widespread across marine systems, yet the ecological and evolutionary drivers shaping nitrifier populations remain largely unknown. The Baltic Sea, a brackish basin with pronounced gradients in salinity, oxygen, nutrients and strong seasonality, is a valuable model environment to investigate these questions. Here, we combined metagenomics and rRNA gene sequencing to characterise the spatiotemporal dynamics and genomic diversity of nitrifiers, alongside physicochemical measurements. Nitrifiers were persistently abundant throughout aphotic waters, with vertical niche partitioning and seasonal peaks in surface waters from late fall to early spring. The seasonal peaks were positively correlated with nitrite, nitrate and diverse other prokaryotes, and negatively correlated with solar radiation and chlorophyll. To probe the genomic basis of these ecological patterns of the numerically dominant nitrifier, we recovered five novel genomes of ammonia‐oxidising archaea through metagenomics of bulk samples and selective enrichments, including the dominant taxon from aphotic depths. Comparative genomics showed high gene conservation, with variation largely in genes linked to interactions with the external environment and nitrogen and phosphorus metabolism between central surface and deep types. Together, our study provides insights into niches of Baltic Sea nitrifiers and begins the process of understanding the mechanisms and functional implications of these patterns.

## Graphical abstract

Nitrifiers in the Baltic Sea show persistent abundance in aphotic waters and late‐fall to winter photic zone increases positively correlated with nutrients and negatively with light and chlorophyll. Through selective enrichment, five novel ammonia‐oxidising archaea genomes were recovered, including the dominant aphotic taxon, which revealed overall high gene conservation and uniqueness related to external environment‐facing features.

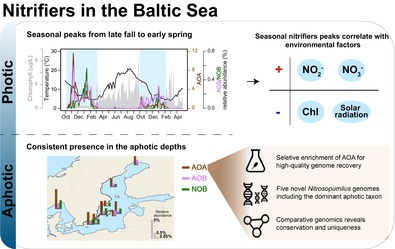

## 1 Introduction

Nitrification is the microbial process whereby reduced nitrogen substrates are oxidised to nitrate, the dominant form of bioavailable nitrogen in the ocean (Gruber 2008). This process plays a crucial role in marine ecosystems and biogeochemical cycling, as nitrogen is a key limiting factor for primary productivity in the surface ocean (Gruber 2008). Despite its importance, nitrification is carried out by a remarkably small number of microbial taxa: ammonia oxidation is performed by ammonia‐oxidising archaea (AOA) (Könneke et al. 2005; Santoro, Richter, and Dupont 2019) and ammonia‐oxidising bacteria (AOB; e.g., *Nitrosomonas*, *Nitrosococcus*), whereas nitrite oxidation is mediated by nitrite‐oxidising bacteria (NOB; e.g., *Nitrospina*, *Nitrospira*) (Tang et al. 2023).

These organisms are ubiquitous across a wide range of natural and artificial ecosystems (Karner et al. 2001; Francis et al. 2005; Leininger et al. 2006; Wuchter et al. 2006; Pachiadaki et al. 2017), and, in the deep sea, nitrifiers are often among the numerically dominant carbon‐fixing organisms, making them key players in sustaining ecosystem function (Ingalls et al. 2006; Reinthaler et al. 2010; Pachiadaki et al. 2017; Bayer et al. 2023). Furthermore, AOA (phylum *Nitrososphaerota*) has been proposed to be the model taxon for deep ocean prokaryotes due to their high abundance and ecological significance (Baker et al. 2024).

Within and between each nitrifier taxon, there is remarkable diversity and variation in their distribution and niches. Although undoubtedly a combination of physiological, ecological and evolutionary factors governs their distributions and dynamics, they are still poorly resolved. Contributing to the differences between families are some long‐recognised traits (Schleper 2010; Qin et al. 2014; Wright and Lehtovirta‐Morley 2023). For example, AOA possess ammonia monooxygenase (AMO) enzymes with a higher affinity compared to AOB, thus driving niche differentiation (Martens‐Habbena et al. 2009; Jung et al. 2022) and allowing them to outcompete AOB in environments with low ammonia availability, and vice versa. There is also trait variability within lineages, for example, different AOA strains have diverse capabilities to take up alternative nitrogen substrates such as urea and cyanate (Alonso‐Sáez et al. 2012; Kitzinger et al. 2019; Qin et al. 2024), degrees of photoinhibition (Guerrero and Jones 1996; Merbt et al. 2012; Qin et al. 2014), and motility (Chain et al. 2003; Schmidt et al. 2004; Bayer et al. 2016), among other factors (Wright and Lehtovirta‐Morley 2023). Together these studies emphasise that the versatility of these populations may have important ecological implications in changing environments.

The Baltic Sea is one of the world's largest brackish environments characterised by a full range of salinity, oxygen concentrations and trophic status driven by variation in pollution and agricultural runoff. It also experiences strong seasonal variability and spans more than 10 degrees of latitudinal gradient. This spectrum of physico‐chemical conditions has led to it being considered a ‘time machine’ for ocean research (Reusch et al. 2018).

In the Baltic Sea, light penetration is generally restricted to the top ~20–30 m (Aarup 2002; Setälä et al. 2005). Below this depth, in the aphotic zone, nitrifiers are active and abundant in the redoxcline where oxygen is still available (Enoksson 1986; Labrenz et al. 2010; Jäntti et al. 2018) and are also present at deeper sub‐oxic depths (Hietanen et al. 2012; Berg et al. 2014, 2015; Wittenborn et al. 2023), with a single AOA represented by 16S rRNA clone sequence GD2 reported to numerically dominate the redoxcline (Labrenz et al. 2010; Hietanen et al. 2012). Although the presence of nitrifiers and nitrification has also been observed in the photic zone (Happel et al. 2018), time‐series dynamics of surface nitrifier populations have not been systematically analyzed.

Here, we utilised basin‐scale Baltic Sea distributions and time‐series sampling to advance knowledge of the niches of nitrifiers at fine phylogenetic resolution. We obtained the genomes of five novel AOA species, including from the dominant deep‐sea strain via selective incubations. Together this allowed us to approach two research questions. (1) What is the distribution, dynamics and diversity of nitrifiers in the Baltic Sea? (2) What genetic differences exist among these organisms and influence their distribution, dynamics and diversity?

## 2 Experimental Procedures

### 2.1 Sample Collection

Spatiotemporal data was collected from across the Baltic Sea. Time‐series data was collected at two locations in the southwest Baltic Sea. In particular, twice‐weekly seawater sampling was carried out for one and a half years from the Southwest Baltic Sea Kiel Fjord (KF, 54°19′48.3″ N 10°09′01.9″ E). For the Kiel Fjord time‐series, seawater (October 2021–May 2023) was collected by bucket and processed within 2 h. Temperature, oxygen and salinity were measured continuously via the Kiel Marine Organism Culture Centre (KIMOCC; https://shorturl.at/bekTW), and temperature and salinity additionally via a handheld sensor (Conductivity portable metre ProfiLine Cond 3110 with Special conductivity measuring cells TetraCon 325 S, WTW), which was used to support KIMOCC data, but not typically used. Weather data were taken from GEOMAR Meteorological Station (https://www.geomar.de/en/service/weather). The full dataset from KF is available via Supplementary Data 1. Additionally, seawater was sampled once monthly via depth‐integrated (1–25 m) sampling at the Boknis Eck time series (BE, 54°30′46.1″ N 10°01′20.5″ E). At Boknis Eck, samples were collected monthly (January 2022–June 2023) from 1, 10, 20 and 25 m via CTD/Rosette. From the Broader Baltic Sea, we collected samples for amplicon sequencing from the Bornholm Basin (5 September 2022, 55°37.50′ N, 15°15′03″ E) and Gotland Basin (3 September 2022, 56°54.90′ N, 19°34.88′ E) via CTD/Rosette. Metagenomic samples collected in this study are listed in Supplementary Data 2.

For nutrient analyses, seawater was frozen at −20°C until measurement in the central analytical laboratory at GEOMAR using a standard colorimetric, continuous flow analyser (QuAAtro 30, SEAL Analytical). Nitrite was determined via reaction with sulphanilamide forming a diazo compound (method Q‐070‐ Rev 7) (Shinn 1941; Bendschneider and Robinson 1952). Nitrite and nitrate (NOx) were determined after reduction on a cadmium coil and measured as nitrite (method Q‐068‐Rev 12) (Grasshoff 1970). Nitrate concentration was calculated as NOx minus nitrite. Phosphate was measured via reaction with molybdate and antimony ions (method Q‐064‐ Rev 8) (Greenfield and Kalber 1954; Murphy and Riley 1962). Silicate was measured via a silico‐molybdate complex reduced to molybdenum blue (Q‐066‐ Rev 5) (Koroleff 1971). Quality control was performed by using certified reference material (CRM) from KANSO TECHNOS CO LTD. Limit of detection was determined by 3* the standard deviation of the lowest standard for each nutrient. For samples with concentrations below the LOD (nitrate: *n* = 21 of 155 and nitrite: *n* = 19 of 155), replacement values were calculated as the LOD/2, as noted in Supplementary Data 1. Replicate nutrient samples, where available, were averaged. As nitrate was calculated as the difference between NOx and nitrite, where nitrite exceeded apparent NOx values, the LOD/2 was used for nitrate.

For chlorophyll a, 200 mL of seawater was filtered on GF/F filters and stored at −80°C, extracted with 90% acetone, and read via fluorometry (Strickland and Parsons 1968).

### 2.2 Filtration, DNA Extraction and Sequencing of Environmental Samples

Seawater samples were vacuum‐filtered under less than 200 mbar pressure through a 0.2 μm pore size filter (Pall, Supor 200 Membrane Disc Filters) and then stored at −80°C until DNA extraction. Just before DNA extraction, 0.25 μL of ZymoBIOMICS Spike‐in Control I (High Microbial Load, D6320) was pipetted directly onto the filter. DNA was extracted from each filter using the DNeasy Plant Kit (Qiagen) with a modified lysis protocol. Briefly, filters were thawed and transferred into screw cap microtubes with mixed glass beads (0.1 and 0.5 mm). Lysis buffer from the kit (AP1) was added, followed by three freeze–thaw cycles using liquid nitrogen and a 65°C water bath. Additional mechanical lysis was conducted via bead‐beating using a Laboratory Mixer (MM 400, Retsch). After lysis, the lysate was transferred to a 96‐well plate for purification via AcroPrep filter plate (Pall Life Sciences). DNA was eluted in Tris‐EDTA and stored at −80°C until amplicon or metagenomic library prep.

For amplicon libraries, 1 ng of DNA (quantified by Quant‐iT PicoGreen dsDNA Assay Kit) was amplified and prepared for sequencing in single PCR reactions with universal V4‐V5 primers 515F and 926R (Parada et al. 2016). The forward primer included an Illumina P5 adaptor sequence (AATGATACGGCGACCACCGAGATCTACAC), a unique 8‐base index with every index differing by at least two base pairs, a forward sequencing primer (ACACTCTTTCCCTACACGACGCTCTTCCGATCT), a random spacer of seven random bases (NNNNNNN) to enhance diversity for the initial base‐pair reads on the Illumina MiSeq followed by the 515F‐Y primer (GTGYCAGCMGCCGCGGTAA). The reverse primer was similarly constructed but with P5 replaced with P7 (CAAGCAGAAGACGGCATACGAGAT) and reverse sequencing primer replaced with (GTGACTGGAGTTCAGACGTGTGCTCTTCCGATCT) and the 926R primer (CCGYCAATTYMTTTRAGTTT). PCR was performed with the KAPA HiFi HotStart PCR Kit (Cat. KK2102, Roche), using 0.4 U of KAPA HiFi HotStart DNA Polymerase, 1× KAPA HiFi Fidelity buffer, 0.3 mM dNTPs and 0.4 μM of each primer. PCR thermocycling consisted of initial denaturation at 95°C for 5 m, followed by 30 cycles of denaturation at 98°C for 20 s, annealing at 55°C for 15 s, and extension at 72°C for 1 m, and a final extension at 72°C for 5 m. Products were cleaned and size‐selected via 0.8× (vol: vol) Ampure XP magnetic beads (Agencourt AMPure XP, Beckman Coulter), or normalised with Charm Biotech Just‐a‐Plate 96 PCR Normalisation plates. For Ampure‐cleaned samples, DNA concentration was quantified with Quant‐iT PicoGreen dsDNA Assay Kit and pooled in equimolar concentration. Prior to sequencing, the pooled DNA was purified again using 0.8× (vol: vol) of the magnetic beads.

For metagenomic libraries, 1 ng of DNA was prepared with Hackflex (Gaio et al. 2022), a modification of the protocol of Illumina DNA Prep (M) Tagmentation Library. Hackflex has been shown to perform well for microbial metagenome sequencing based on mock community analysis and comparison to other library preparation methods (Goldman et al. 2024). Following Gaio et al., DNA was tagmented in 50 μL reactions and then amplified with Phusion Hot Start Flex DNA Polymerase (New England Biolabs, M0535) and amplified with forward and reverse primers that consisted of P5 or P7 adapters, 8‐base indexes, and partial overhangs to the transposome adapter TCGTCGGCAGCGTC or GTCTCGTGGGCTCGG, respectively. PCR was carried out with an initial denaturation at 98°C for 30 s, followed by 12–16 cycles of: 98°C for 10 s, 62°C for 30 s, 72°C for 30 s and 72°C for 5 m for a final extension. After PCR, products underwent 0.6× size selection via Ampure XP magnetic beads. The products were then re‐evaluated using a bioanalyzer, followed by an additional 0.8× bead clean‐up to remove short DNA fragments.

All sequencing was performed at the Competence Centre for Genomic Analysis (CCGA) Kiel, with amplicon libraries sequenced on MiSeq (2 × 300 bp) and metagenomic libraries on Novaseq (2 × 150 bp).

### 2.3 Selective Enrichment and Sequencing of AOA

In an effort to selectively incubate and enrich AOA from natural seawater, we used an Archaeal medium adapted from (Santoro and Casciotti 2011) with modifications. In brief, surface water was collected from the Kiel Fjord and then aged. This water serves as the base of the medium. Incubations were initially established using 0.2 μm filtered but non‐autoclaved seawater to avoid potential adverse effects on organic content, and the media were amended with 100 μmol/L NH_4_Cl, 1 mL/L chelated trace element solution, 15 μmol/L KH_2_PO_4_, and 50 μg/mL ampicillin. However, *Ca*. N. eckernfoerdensis and *Ca*. N. balticoprofundus were directly incubated with autoclaved seawater amended with the same components. In incubations that showed increases to 70–100 μM nitrite, transfers were made into autoclaved seawater media with the same amendments. To reduce the diversity and abundance of bacteria, two selected strains were further supplemented with 50 μg/mL of streptomycin and kanamycin. The incubations were maintained at room temperature, except for the dominant deep Baltic Sea strain (*N. balticoprofundus*), which was incubated at 10°C.

In order to molecularly characterise the organisms present in incubations with accumulated nitrite, at the time of transfer, 1–2 mL of the incubation was filtered through a 0.2 μm Supor filter at < 200 mbar pressure and stored at −80°C. Then, the same DNA extraction as above DNeasy Plant Kit (Qiagen) was followed, except instead of DNA purification occurring on plates, it was performed in individual tubes. For rRNA gene amplicon sequencing library preparation, we followed the same protocol described for environmental samples but used 1 μL of DNA extract with 30 PCR cycles. Incubations showing distinct ASVs were selected for metagenomic sequencing using the Hackflex protocol (12–16 PCR cycles).

### 2.4 Amplicon and Metagenomic Data Processing

For rRNA gene amplicon data, the primers 515F and 926R were trimmed from raw reads using Cutadapt v4.4 (Martin 2011). 16S and 18S amplicons were split with bbsplit.sh (Bushnell 2016) by comparison to SILVA 132 (Quast et al. 2013) and PR2 (Guillou et al. 2013). The subsequent analysis focused exclusively on prokaryotic reads, using QIIME2 v2022‐2 and/or v2022‐11 (Bolyen et al. 2019). Forward and reverse reads were based on sequence quality scores from FastQC v0.12.1 (Andrews 2010) and denoised to amplicon sequence variants (ASVs) with DADA2 (Callahan et al. 2016). Taxonomic classification was performed on ASVs with VSEARCH (Rognes et al. 2016) and the SILVA 138.1 database (Quast et al. 2013). Individual sequencing runs were analyzed separately and subsequently merged, as recommended (Callahan et al. 2016). Chloroplast, mitochondrial and unassigned reads were then removed using the taxa filter‐table function in QIIME2. For calculating relative abundance data, reads originating from the spiked‐in controls were manually identified and removed from relative abundance data, and the count of each ASV within each sample was calculated by division by the total number of remaining prokaryotic sequences in each sample. Samples with low sequencing depth (< 900 reads) were filtered out.

To estimate gene copies of ASV in each sample, we extrapolated their read counts to gene copies by comparing to the two exogenous bacterial standards from ZymoBIOMICS Spike‐in Control I (High Microbial Load) via:

$${\mathrm{ASV}}_{\mathrm{ij}}=\frac{R_{\mathrm{ij}}C_{s}}{R_{\mathrm{sj}}V_{j}}$$

where *ASV* is the number of gene copies of each taxon (*i*) in a sample (*j*), *R*_*ij*_ is the number of *ASV* reads, C_s_ is the number of genomic spike‐in rRNA gene copies, *R*_*sj*_ is the number of reads of spike‐in *ASV*s, and *V*_*j*_ is the volume filtered. Samples with fewer than 10 spike‐in reads were excluded from the estimation to avoid unreliable extrapolation. The Spearman correlation coefficients between estimated cell abundances and relative abundances for common nitrifiers ASVs were calculated using R (cor.test, method = ‘spearman’) (Supplementary Data 3).

For metagenomic analysis, paired‐end reads were quality‐filtered with Trimmomatic v0.39 (Bolger et al. 2014) by truncating at the first base with a quality score below three, followed by further truncation if the moving average quality score across 25 base pairs fell below 30 (settings: LEADING:3 TRAILING:3 SLIDINGWINDOW:25:30 MINLEN:50). The resulting paired reads were then assembled with SPAdes v3.13.0 (Bankevich et al. 2012), with k‐mer sizes (21, 33, 55, 77, 99, 127) and the ‘‐sc’ option. For downstream analysis, only contigs exceeding 5000 base pairs were retained. Quality‐filtered paired reads were mapped back to contigs with Bowtie2 v2.3.5 (Langmead and Salzberg 2012) for each respective sample. Enrichments were processed individually. All sets of metagenomic reads were mapped to the individual sample assemblies to enable binning while reducing the potential for chimeric contigs. For enrichments, the binning process was conducted using the Anvi'o v8 (Eren et al. 2015) interactive interface (Eren et al. 2015, 2021; Delmont and Eren 2018), leveraging information such as tetranucleotide frequency, GC content and contig coverage. For environmental samples, the data were first automatically binned with Metabat (Kang et al. 2015) and then refined using Anvi'o (Eren et al. 2015). Environmental metagenomic bins were then de‐replicated with dRep (Olm et al. 2017). The quality of all resulting bins from incubated AOA and environmental samples was evaluated using CheckM v1.2.2 (Parks et al. 2015). Finally, taxonomic classification for all bins was performed using GTDB‐Tk v2.1.1 (Chaumeil et al. 2019). Genomes were assigned Candidatus species names in accordance with SeqCode requirements (Hedlund et al. 2022) as detailed in the Protologue in Appendix A. One genome (Bin_54a), which did not meet the completion threshold, was left with a placeholder name.

### 2.5 16S rRNA Gene Phylogenetics and Phylotyping From Metagenomes

rRNA gene reads were extracted via SortMeRNA (Kopylova et al. 2012) (‐e 0.0001) from 15 metagenomes first reported here as well as from 169 public metagenomes (Hugerth et al. 2015; Asplund‐Samuelsson et al. 2016; Alneberg et al. 2018, 2020) (Supplementary Data 2), based on the smr_v4.3_sensitive_db.fasta database. The taxonomic classification of the reads was used to select *Nitrosopumilus*, Nitrospinaceae, Nitrospiraceae, Nitrosomonadaceae and Nitrosococcaceae. Relative abundances were calculated from the number of reads classified as these respective groups divided by the total amount of reads predicted to be 16S rRNA gene reads in general. We chose to focus on *Nitrosopumilus* within Nitrosopumilaceae, as no other Nitrosopumilaceae were abundant in the Baltic Sea.

In order to phylogenetically classify (phylotype) the 16S rRNA gene amplicons and 16S rRNA gene reads from metagenomes for the groups above, we constructed phylogenetic trees of the 16S for *Nitrosopumilus*, *Nitrospinaceae*, *Nitrospiraceae*, *Nitrosomonadaceae* and *Nitrosococcaceae*. The phylogenetic trees were assembled from a variety of resources. First, we selected 16S rRNA genes from the Greengenes2 (McDonald et al. 2024) database. Second, we assembled 16S rRNA gene reads from quality‐filtered metagenomes derived from Baltic Sea samples with MATAM (Pericard et al. 2018). In the case of *Nitrosopumilus*, we also extracted 16S rRNA reads from reference genomes as well as those assembled from our own data. In all cases, the 16S rRNA genes longer than 800 bp were clustered with VSEARCH (Rognes et al. 2016) at 0.995 sequence similarity, selecting the centroid as the representative sequences. Sequences were then aligned with MUSCLE v3.8.1551 (Edgar 2004) default settings, trimmed with trimAI (Capella‐Gutiérrez et al. 2009) (‐gt 0.1), and phylogenetic trees constructed with IQ‐TREE (Nguyen et al. 2015) (‐m GT ‐B 1000 ‐alrt 1000). The resulting trees were clustered by TreeCluster.py (Balaban et al. 2019) (‐t 0.01, ‐s 0.80 ‐m max_clade) in order to group clades. ASV sequences as well as those predicted by SortMeRNA were then placed on these trees with epa‐ng (Barbera et al. 2019) (‐‐filter‐min‐lwr 0.01). Resulting jplace files were processed with gappa (Czech et al. 2020) and converted to csv with guppy (Matsen et al. 2010). In the downstream analysis, in order to restrict the analysis to the most confident detections, only samples with > 100 total prokaryotic 16S reads were included, phylotypes with fewer than 250 total reads across all samples were excluded, and for a given sample, taxa with less than 25 reads were considered absent.

### 2.6 Phylogenomics and Comparative Genomics of AOA

*Nitrosopumilus* genomes considered as species representatives were retrieved from GTDB data v214.1 (Parks et al. 2020) using GToTree v1.8.1 (Lee 2019). Each reference genome underwent a quality assessment using CheckM v1.2.2 (Parks et al. 2015) to ensure a minimum of 80% completion and less than 5% redundancy. From the initial set of reference genomes, those showing > 95% average nucleotide identity with genomes from this study were excluded (*n* = 2), because in both cases they had lower genome completion and/or lower N50. The final dataset encompassed 60 genomes, including 52 *Nitrosopumilus* representatives from GTDB, seven from this study (Supplementary Data 4), and *Ca*. Nitrosopelagicus brevis as an outgroup. For incomplete genomes, estimated genome sizes were calculated using a combination of completion and redundancy estimates (Nayfach et al. 2019).

The phylogenomic tree was constructed by GToTree v1.8.1 (Lee 2019), with the archaeal single‐copy genes set (76 genes). The analysis pipeline included gene prediction using Prodigal v2.6.3 (Hyatt et al. 2010), target gene identification with HMMER3 v3.3.2 (Eddy 2011), individual gene alignment using muscle 5.1.linux64 (Edgar 2004), and alignment refinement with trimAl v1.4.rev15 (Capella‐Gutiérrez et al. 2009) before concatenation. The phylogenetic tree was estimated using IQ‐TREE (Kalyaanamoorthy et al. 2017; Hoang et al. 2018; Minh et al. 2020), supported by 1000 bootstrap replicates. For functional gene annotation, selected gene sets for ammonia oxidation (amoABC), UV repair (uvrABC), osmoregulation (ectoine, ectABC), phosphate transport (pstABC), urease (ureABCDEFG), DNA phosphorothioation (dnd) and motility (archaella), and 3‐hydroxypropionate/4‐hydroxybutyrate (3HP/4HB) cycle related genes (Walker et al. 2010) were manually identified from various *Nitrosopumilus* species, with gene IDs manually gathered from NCBI and UniProt (Supplementary Data 5). These genes were downloaded using the ‘ncbi datasets’ tool and analyzed using NCBI BLAST+ (Camacho et al. 2009). BLAST results were filtered (*e*‐value > 1e‐5 and bitscore > 100) and summarised in a gene presence‐absence table. The presence of each functional category/pathway (Figure 6) was determined based on whether more than half of the related genes were detected. The tree was visualised with R 4.2.2 using ggtree v3.6.2 (Yu et al. 2017), and gene patterns and metadata were visualised using ggplot2 (Wickham 2016a). The alignment of these elements next to the tree was achieved using the aplot package (Yu 2023) and other tools to process tree data (Paradis and Schliep 2019; Wang et al. 2020; Yu 2022).

For pangenomics, to compare all the genomes within the genus *Nitrosopumilus*, we employed Anvi'o v8 (Delmont and Eren 2018; Eren et al. 2021). For each genome, databases were created and annotated with important gene sets (rRNA and single‐copy core genes of taxa) using built‐in databases, with initial functional assignments using the Clusters of Orthologous Genes (COGs) database (Tatusov et al. 2000). Protein sequences were aligned using BLASTP (Camacho et al. 2009) and clustered using MCL (van Dongen and Abreu‐Goodger 2012) with an inflation value of 10 for fine‐scale cluster division. To analyze gene distribution patterns, we classified the gene clusters into four categories based on the number of genomes contributing genes to each cluster. Given the inclusion of metagenome‐assembled genomes (MAGs) to pangenomics, we adopted more flexible classification criteria for gene clusters (Li and Yin 2022): core (genes present in 51–60 genomes, ≥ 85%), semi‐core (28–50 genomes, 47%–83%), flexible (3–27 genomes, 5%–45%) and rare (1–2 genomes, < 5%). The gene cluster summary was exported, and additional functional annotations were obtained using eggNOG‐mapper (Cantalapiedra et al. 2021). These annotations were integrated with the cluster summary data to examine how genes from each COG20 functional category were distributed among core, semi‐core, flexible and rare gene cluster categories.

We used the 60 representative genomes as references to map the metagenomic data at 0.95 similarity using BBMap (Bushnell 2016). The ‘trimmed means’ were then computed with CoverM (Aroney et al. 2025) genome default settings: ‐‐contig‐end‐exclusion and –min‐covered‐fraction of 0.25.

### 2.7 Statistical Analyses of Environmental Data

To analyse the correlation between the relative abundance of each microbe and environmental parameters, eLSA v1.0.2 (Xia et al. 2011) was used to calculate the time‐lagged correlations using data from Kiel Fjord. Samples lacking 16S rRNA amplicon data or with low reads (< 900) were excluded, and ASVs observed in more than 5% of samples were retained (i.e., at least 9 out of 161 samples). For environmental data, missing values were manually linearly interpolated within the time series. The analysis was run with a maximum time lag of six sample points (~3 weeks), and significance values were calculated with the ‘mixed’ method using 1000 permutations. To analyze the correlation between abundant nitrifiers and environmental parameters, key ASVs and relevant environmental parameters were extracted from the eLSA results and visualised as a heatmap using R (v4.2.2) with ggplot2 (Wickham 2016b), with hierarchical clustering performed using Ward's method. The resulting correlation network was filtered to retain only strong and significant correlations (correlation > 0.7, *p* ≤ 0.001, *q* ≤ 0.001). The filtered network was visualised using Cytoscape (Shannon et al. 2003), summarising important nodes and edges associated with nitrifiers.

### 2.8 Sample Overview

A comprehensive overview of all datasets used in this study, including newly generated and publicly available metagenomes and amplicon datasets, is summarised in Supplementary Data 6.

## 3 Results

### 3.1 Basin‐Scale Distributions of Nitrifiers

**FIGURE 1:**
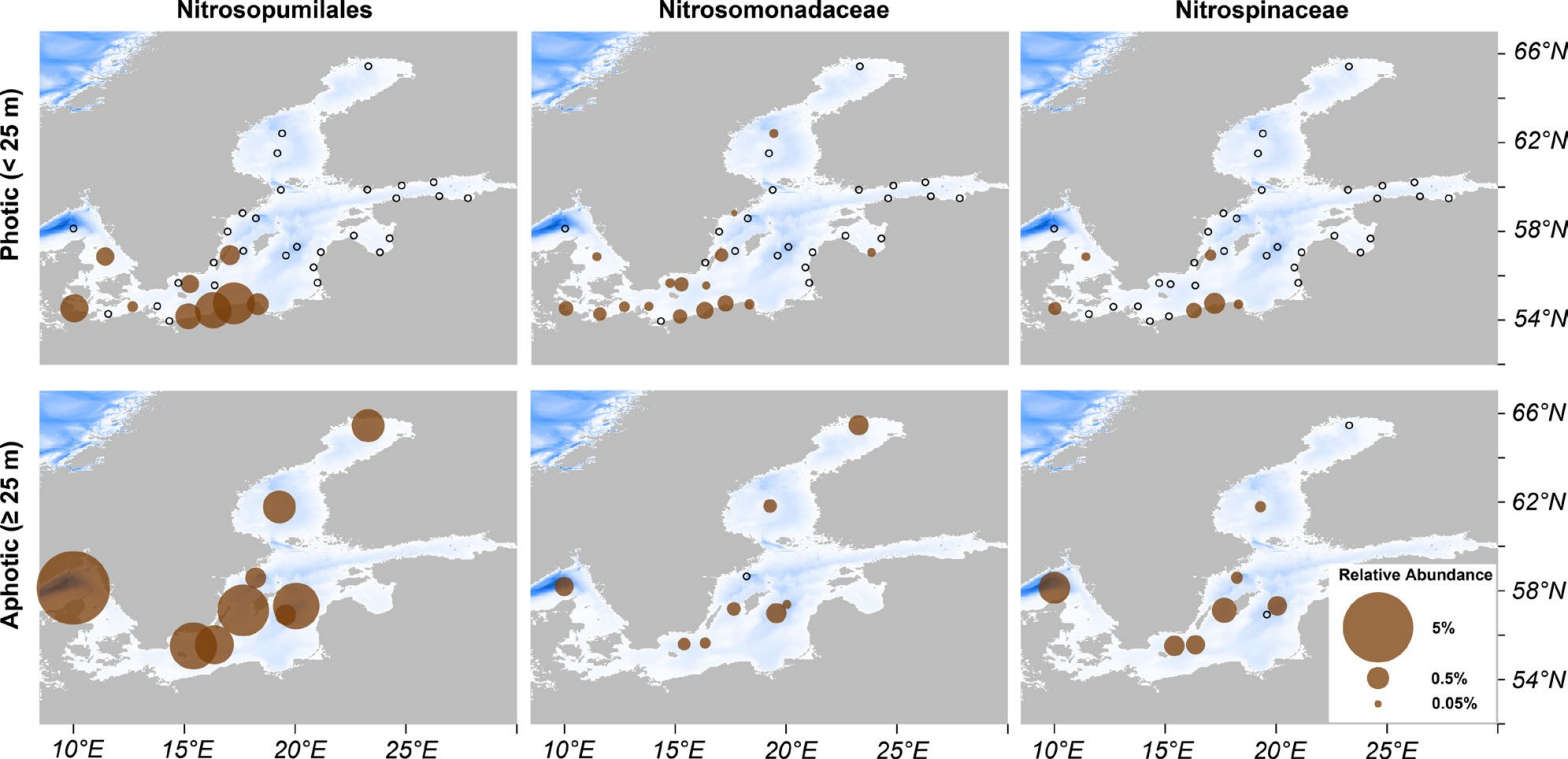
Distributions of nitrifiers across the entire Baltic Sea. Relative abundance of nitrifiers rRNA gene sequences was extracted from metagenomic datasets and classified using SortMeRNA. The pie size indicates the average relative abundance of each family at a given location (samples within 1° of latitude and longitude are averaged). The top row shows samples collected from the photic depths (0–25 m), and the bottom row shows aphotic samples (≥ 25 m). Open black circles indicate no detection. Background shading (white and blue) represents water column depth. Most metagenomic data utilised here was previously published (Hugerth et al. 2015; Asplund‐Samuelsson et al. 2016; Alneberg et al. 2018, 2020).

To gain an overview of the distributions and diversity of nitrifiers on a basin‐wide scale, we surveyed 184 water column metagenomes, including 15 newly generated in this study and 169 from previously published datasets (Hugerth et al. 2015; Asplund‐Samuelsson et al. 2016; Alneberg et al. 2018, 2020). Overall, Nitrosopumilaceae had the highest relative abundance among nitrifiers, comprising 0.53% of prokaryotic rRNA gene reads (Figure 1), followed by 0.12% for both Nitrosomonadaceae and Nitrospinaceae. Nitrosopumilaceae, Nitrosomonadaceae and Nitrospinaceae were detected in 100%, 89% and 78% of the 1° × 1° spatial bins that contained aphotic‐zone samples (herein, ≥ 25 m). In contrast, their detection in photic‐zone bins (< 25 m) was lower (24%, 42% and 15.8%, respectively) (Figure 1). Taxa with only marginal presence, such as Nitrosococcaceae and Nitrospiraceae, were excluded from further analysis to focus on the three major nitrifier groups (Supplementary Data 2).

Beyond the overall abundance patterns, we found that the predominant phylotypes of nitrifiers generally differed in their abundances between photic and aphotic depths (Figure S1A). In the photic zone, a single Nitrosopumilaceae phylotype related to *Ca*. N. limneticus (Klotz et al. 2022) dominated, accounting for an average of 97% of the family across all photic zone samples (Figure S1A). The aphotic zone, however, contained a more mixed AOA assemblage. The limneticus‐related phylotype remained the most relatively abundant (averaging 58%), but another phylotype related to *N. oxyclinae* HCE1 (Qin et al. 2017) and the GD2 cluster (Labrenz et al. 2010) was also common (averaging 37%) (Figure S1B,C). Similarly, AOB and NOB also showed vertical niche partitioning, with certain phylotypes favouring surface layers and others more abundant in aphotic waters (Figure S1B,C).

To complement the basin‐wide metagenomic observations, we utilised universal rRNA gene amplicon sequencing from two depth profiles in the Baltic Sea proper in September 2023 (Figure S2). Consistent with previous results, AOA were again dominant in aphotic waters, with the most abundant ASV (*Nitrosopumilus* a) showing 99.7% similarity (376/377 bp) to the 16S rRNA gene of the GD2 cluster. In the Bornholm Basin, *Nitrosopumilus* ASVs accounted for 2.2%–16.7% of total 16S rRNA reads between 25 and 70 m, whereas AOB and NOB each remained ≤ 1% and ≤ 2.8%, respectively (Figure S3).

Both stations showed clear vertical structuring of nitrifier communities, but the pattern was especially pronounced in the Gotland Basin, where the community exhibited a sharply peaked vertical structure. AOB reached their maximum at 25 m (90% of nitrifier reads), declined at 50 m (69.9%), and were nearly absent at 150 m (0%), where *Nitrosopumilus* ASVs again dominated (6.3% of total reads and 98% of nitrifier reads). Notably, individual ASVs within each group also showed distinct depth preferences, indicating ecological differentiation even among closely related strains and supporting the vertical niche partitioning inferred from the metagenomic analysis.

### 3.2 Seasonality of Nitrifiers and Environmental Parameters

Given the sporadic detection of nitrifiers in photic waters and seasonal dynamics of nitrifiers previously reported in other marine ecosystems, we hypothesised that seasonal dynamics in nitrifier communities may occur in the Baltic Sea.

**FIGURE 2:**
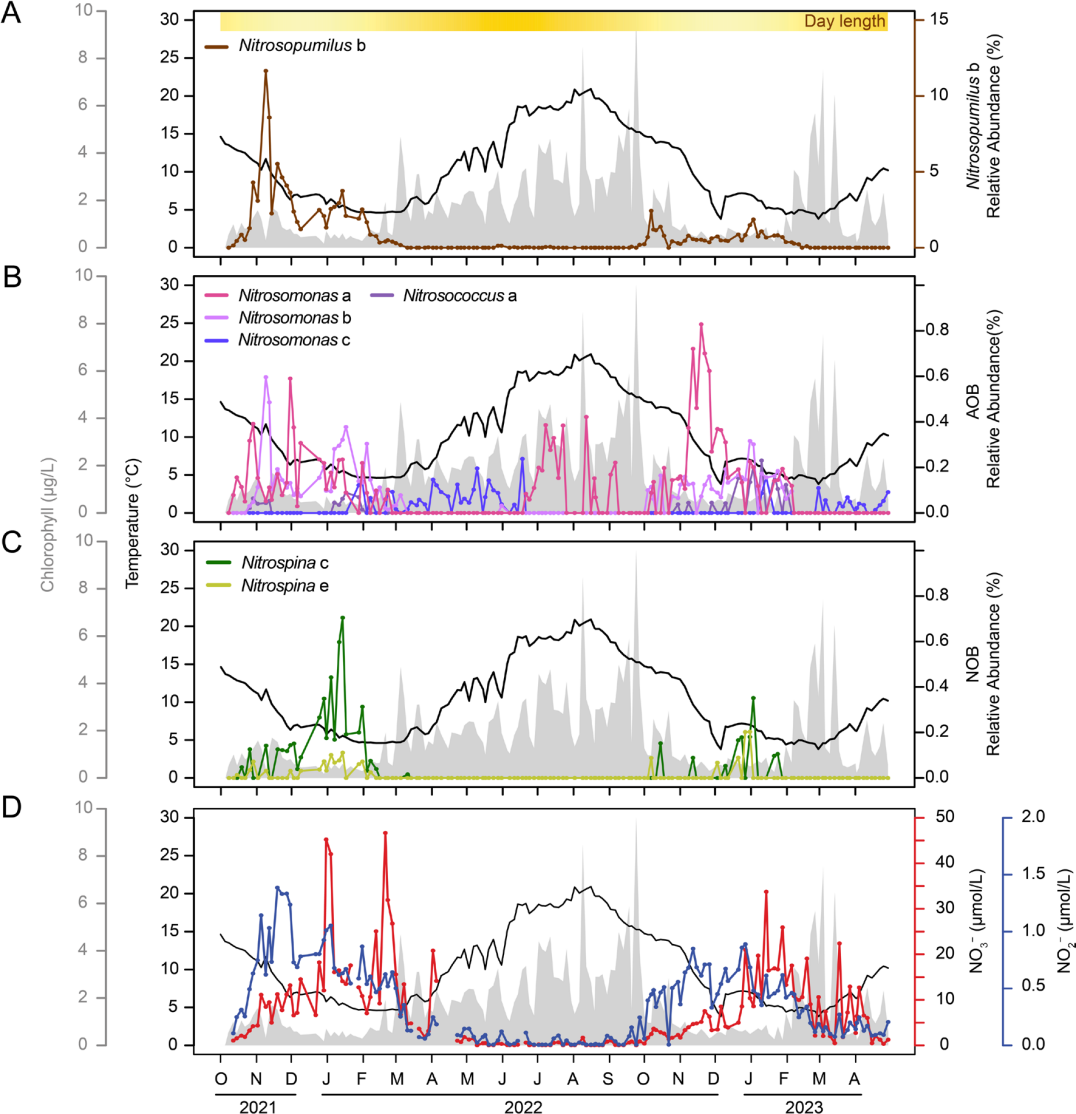
Time‐series dynamics of nitrifier ASVs and inorganic nitrogen at the Kiel Fjord Time‐series Station. Based on 16S rRNA gene amplicon analysis, the top three panels show the relative abundances (among all prokaryotes) of nitrifier ASVs: (A) AOA, (B) AOB and (C) NOB. Coloured lines represent individual ASVs. (Panel D) displays concentrations of NO_2_^−^ (blue) and NO_3_^−^ (red). The yellow bar at the top of panel A indicates day length, with more intense yellow indicating longer daylight periods. Overlaid black lines indicate temperature, and grey shading shows chlorophyll concentration. One AOA ASV (*Nitrosopumilus* b) dominated the AOA community, with 58‐fold higher abundance than the second most relatively abundant AOA ASV (not shown). AOA and NOB peaks occurred in late‐fall to winter, whereas AOB were more evenly distributed throughout the year.

To address this, we collected surface water samples twice weekly from the Kiel Fjord in the southwest Baltic Sea between October 2021 and April 2023 (Figure S2). Utilising universal rRNA gene amplicon sequencing, we observed pronounced seasonality in the relative abundance of nitrifier communities, especially AOA and NOB (Figure 2). We found that AOA dominated in relative abundance, averaging 0.86% of total prokaryotic 16S rRNA reads across the time series, compared to 0.07% and 0.04% for AOB and NOB. Within AOA, a single ASV (*Nitrosopumilus* b) accounts for 96.7% of all AOA reads (Figure 2A). Across both years, *Nitrosopumilus* b showed strong seasonality, peaking in October–November and remaining high until March (1.22% average relative abundance from October to March vs. 0.009% from April to September). Interannually, overall AOA abundance was 3.7‐fold higher in winter 2021–2022 compared to the following winter.

Beyond AOA, other nitrifiers were also increased during late fall and winter in the Kiel Fjord surface waters. However, unlike the surface AOA dynamics, AOB were more diverse and consistently observed even in summer, showing a clear succession of different AOB ASVs over time. *Nitrosomonas* c appeared in spring and persisted into early summer before disappearing, after which *Nitrosomonas* a re‐emerged and persisted through autumn and winter together with other winter‐associated AOB ASVs (Figure 2B). In contrast, NOB increased together with AOA and AOB during late fall and early winter, but their peak occurred later, reaching maximal relative abundances in mid winter, which is consistent with the expected temporal lag in the nitrification pathway (Figure 2C).

We also estimated cell abundances of nitrifiers using a spike‐in approach with a known amount of exogenous bacterial cells (see Experimental Procedures). The estimated cell abundances of all dominant nitrifier ASVs had significant positive correlations to their respective relative abundances (Spearman's *ρ* = 0.92–0.996, all *p* < 1E‐50, *n* = 124, Supplementary Data 3), largely mirroring the same seasonal trends (Figure S4). However, due to limited spike‐in recovery in some samples, we focused on relative abundance for subsequent analyses.

To assess whether the seasonal dynamics observed in Kiel Fjord were consistent across sites in the southwestern Baltic Sea, we additionally analyzed rRNA gene amplicon data from the Boknis Eck time‐series station (Figure S2), which spans a 25 m water column and was sampled monthly during the same time period as the surface sampling in the Kiel Fjord. At 1 m depth at Boknis Eck, nitrifier communities exhibited seasonal patterns similar to those in Kiel Fjord, including a winter peak and a decline in the second year (Figure S5). In the deeper depths (20 and 25 m) at Boknis Eck, nitrifiers showed higher overall relative abundances and more persistence overall (Figure S5), suggesting reduced temporal variability under lower light conditions.

### 3.3 Correlations With Environmental Parameters

**FIGURE 3:**
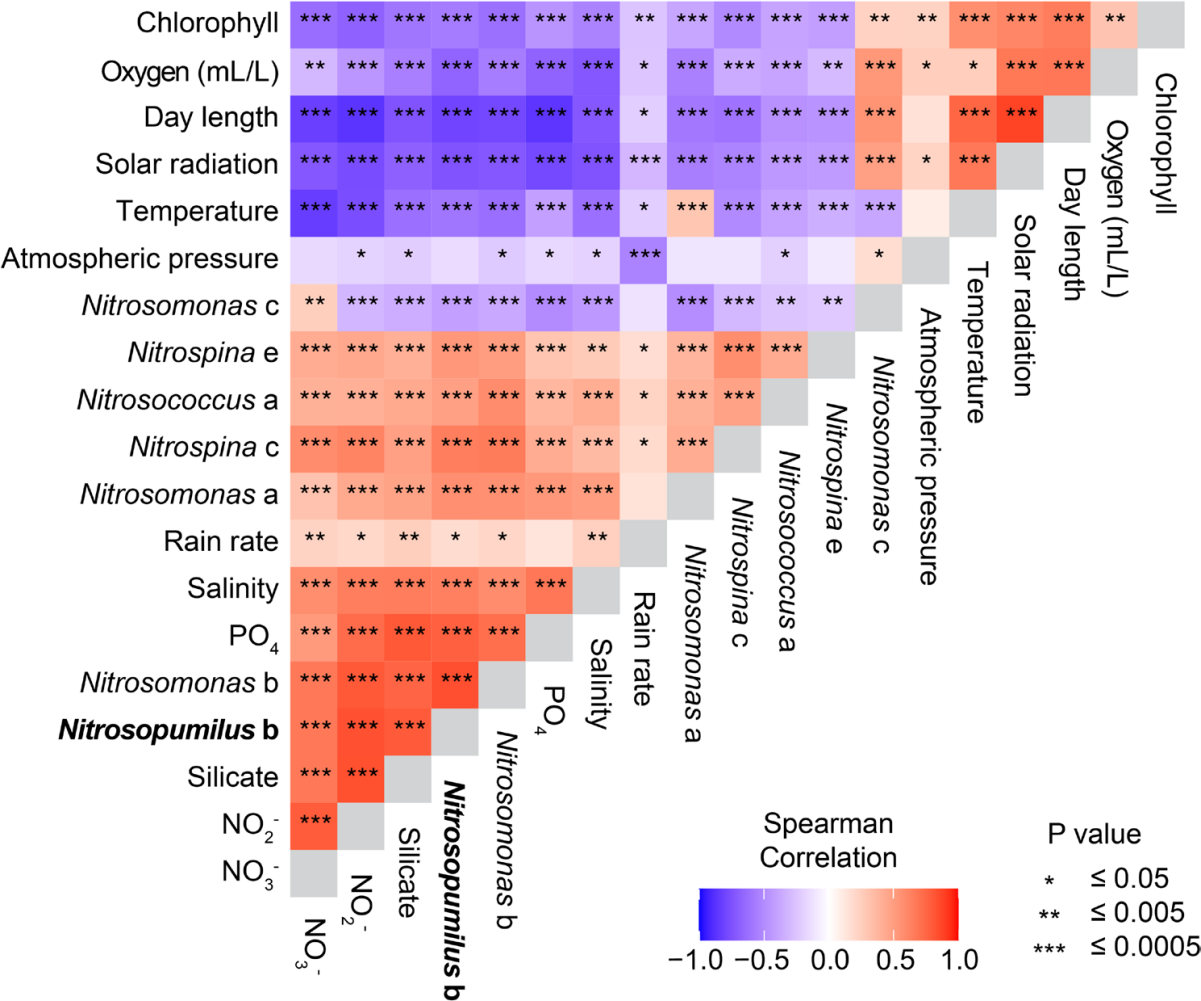
Correlations between nitrifier ASVs and environmental parameters at the Kiel Fjord Time‐series Station. Heatmap showing the correlation between ASVs of nitrifiers (AOA, AOB and NOB) and environmental parameters over 1.5 years. Time‐shifted Spearman's correlation coefficients were computed using extended Local Similarity Analysis (eLSA) to assess correlations. Colours indicate the strength and direction of the correlation, with asterisks denoting levels of statistical significance.

To gain further insight into the environmental drivers potentially shaping seasonal nitrifier dynamics, we examined high‐resolution, twice‐weekly time series from Kiel Fjord. In general, nitrifiers had a strong positive correlation with inorganic nitrogen concentrations, where nitrite peaked in late fall (Figure 2D) and nitrate in late winter (Figure 2D), resulting in a strong positive correlation with the peak abundances of nitrifiers (Figure 3). On the other hand, nitrifiers had negative correlations with several seasonal, co‐varying factors, including temperature, chlorophyll, solar radiation, day length, oxygen and positive correlations with inorganic nutrient concentration (Figure 3). One AOB ASV (*Nitrosomonas* c) deviated from this trend, showing distinct environmental correlations compared to other nitrifiers (Figure 3).

**FIGURE 4:**
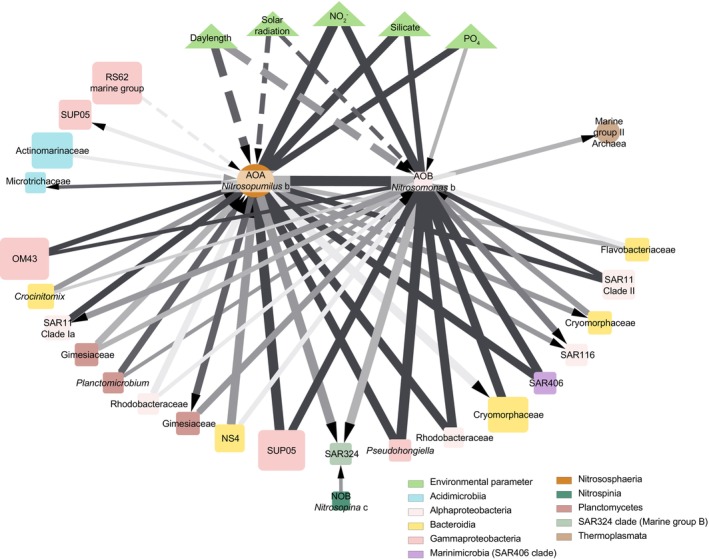
Network analysis of nitrifiers, associated microbial taxa and environmental parameters from the Kiel Fjord Time‐series Station. Network edges are based on time‐shifted Spearman's correlation coefficients calculated using extended Local Similarity Analysis (eLSA). Nodes represent taxa (squares: Bacteria; circles: Archaea) or environmental parameters (triangles), with node size proportional to their mean relative abundance. Edges show significant correlations (|*ρ*| > 0.7, *p* ≤ 0.001, *q* ≤ 0.001) with a lag tolerance of up to 3 weeks. Edge colour reflects temporal relationships, with darker colours representing concurrent correlations and lighter colours indicating increasing time lags. Solid and dashed edges indicate positive and negative correlations, respectively. Arrows denote the direction of delayed responses.

To further quantify relationships between nitrifiers, environmental conditions and other prokaryotic taxa, we conducted a correlation network analysis using the same twice‐weekly Kiel Fjord time‐series dataset (Figure 4). In addition to strong correlations between ammonia oxidizers and nitrite, we identified significant positive relationships with diverse prokaryotes from eight different Orders, including prevalent community members such as SUP05, Cryomorphaceae, NS4, SAR406, Planctomycetes, SAR324 and SAR11 Clade II. The sole negative correlation observed was between the dominant AOA and the summer‐abundant Burkholderiaceae RS62 marine group.

### 3.4 Selective Enrichment and Eco‐Genomics of AOA

Our observations consistently indicated AOA as the numerically dominant nitrifiers across temporal and spatial scales in the Baltic Sea, with strong niche partitioning between the dominant types across locations and depth. Thus, to advance our understanding of these abundant taxa, we set out to obtain representative genomes.

Given that AOA genomes are generally challenging to recover from bulk metagenomic and single‐cell sequencing studies (Santoro, Kellom, and Laperriere 2019), we carried out selective enrichment of AOA from Baltic Sea water samples. To enrich AOA, we transferred Baltic Sea water samples into a medium enriched with ammonium (100 μM), other elements, and antibiotics (see Experimental Procedures). We assessed for evidence of ammonia oxidation via nitrite accumulation, and enrichments that exhibited nitrite accumulation (> 70 μM) were transferred into fresh media. The time required for nitrite to reaccumulate again varied across incubations, ranging from 2 weeks to 4 months (Figure S6). We performed rRNA gene and metagenomic sequencing to confirm the presence of AOA and assess the overall diversity of the enrichments exhibiting nitrite accumulation.

**FIGURE 5:**
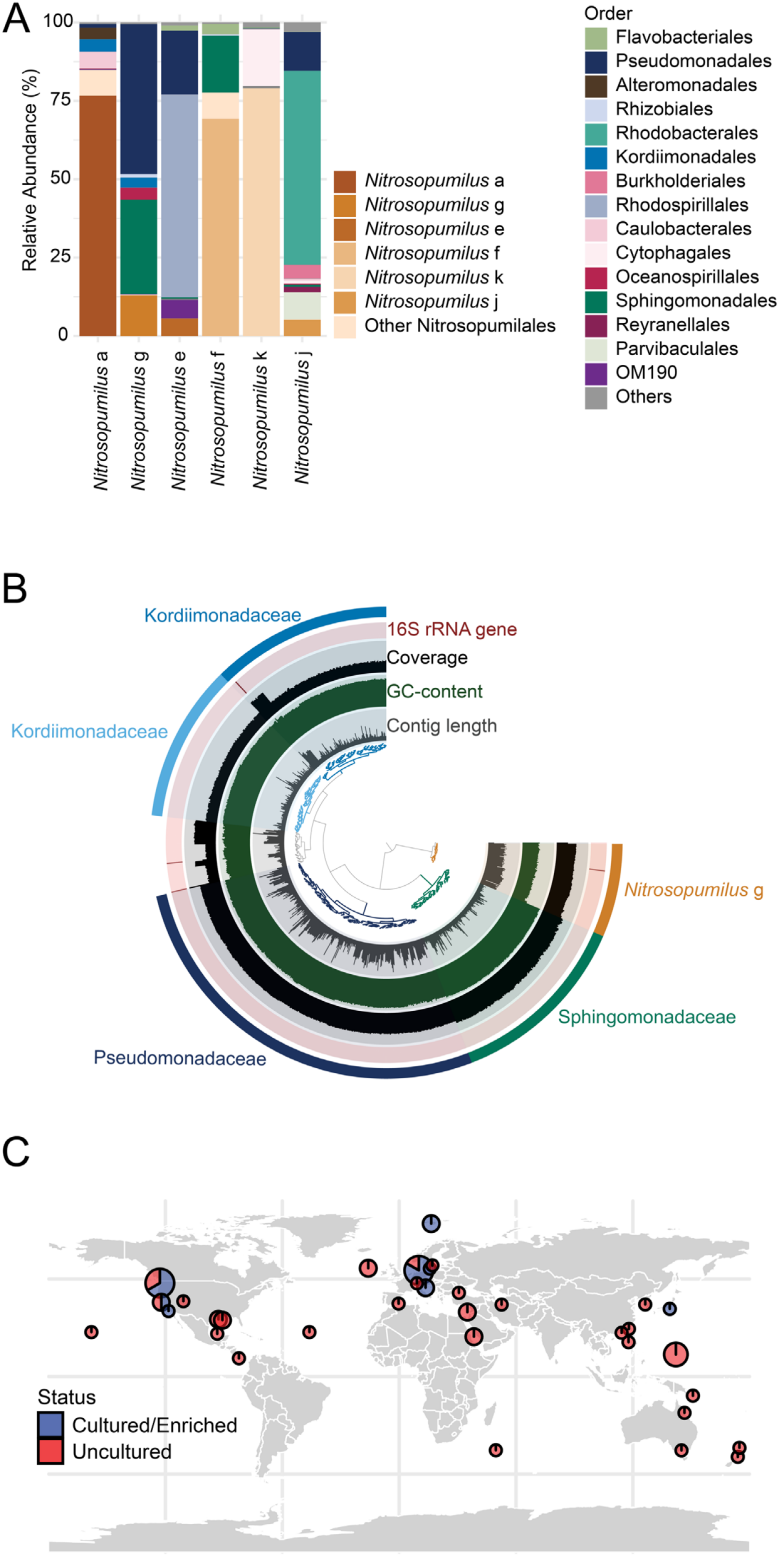
Selective enrichment, genome binning and global distribution of AOA genomes. (A) Microbial community composition across different selective enrichments, showing increased relative abundance of *Nitrosopumilus* ASVs (brown shades), whereas other bacterial taxa are shown at the order level. *Nitrosopumilus* f (*Ca*. N. phosphorothiolatus) and *Nitrosopumilus* k (*Ca*. N. balticomotus) cultures show reduced bacterial diversity due to additional antibiotic treatment. (B) Representative manual binning of a selective enrichment via metagenomic assembly in Anvi'o. The archaeal bin is highlighted by its distinct GC content and coverage patterns. Each column represents, from outside to inside, the presence of 16S rRNA genes, coverage, GC content and contig length. (C) Global distribution of representative *Nitrosopumilus* genomes from GTDB, along with genomes first described in this study. Among 59 genomes analysed, 55 genomes are shown here, excluding those lacking sampling location metadata.

rRNA gene sequencing demonstrated that AOA were the only nitrifiers present in the enrichments, making up 5%–80% of the prokaryotic community (Figure 5A). The selective conditions of the incubation led AOA genomes to stand out starkly in the metagenomic sequencing results (Figure 5B), enabling manual curation of their assemblies based on tetra‐nucleotide frequency, GC content and coverage. From these assemblies, we recovered six high‐quality, near‐complete genomes of *Nitrosopumilus* candidate species, including five novel species and one genome closely related (95.3% ANI) to a previously reported genome (GCA_008080815.1). We assigned Candidatus species names for the five novel genomes (See Protologues in Appendix A). These genomes expand the known genomic diversity of marine AOA and complement 54 high‐quality *Nitrosopumilus* representatives in the GTDB database (v214.1) (Figure 5C).

Among the novel AOA genomes, one corresponded to the dominant deep Baltic Sea strain and originated from an incubation initiated with water collected at 63 m depth in the Bornholm Basin (Figure S2). This genome matched the dominant aphotic‐zone AOA ASV, *Nitrosopumilus* a, with 100% 16S rRNA identity, and also shared 99.7% identity with the GD2 cluster previously reported from the Baltic redoxcline (Labrenz et al. 2010). Based on its consistent enrichment under selective incubation and dominance in aphotic Baltic Sea communities, we propose the name *Candidatus* Nitrosopumilus balticoprofundus for this species.

Although we were unable to enrich a strain corresponding to the dominant surface AOA from the southwestern Baltic Sea (*Nitrosopumilus* b), we recovered a representative MAG for this strain through bulk metagenomic approaches. We propose the name *Ca*. Nitrosopumilus sp. balticolittoralis for this MAG, reflecting its dominance in the coastal, shallow (littoral) waters of the Baltic Sea. *Ca*. N. sp. balticolittoralis shares 98.5% ANI with a formally undescribed MAG from the Black Sea (GCA‐14384445). Its ammonia monooxygenase alpha subunit (amoA) gene shows 98.6% nucleotide identity to HQ713535.1, which is the closest known reference to the dominant AOA (‘aOTU1’) reported in surface waters across both the northern (Öre Estuary) and southern Baltic Sea (Bay of Gdańsk) (Happel et al. 2018), initially suggesting that *Ca*. N. balticolittoralis, or its close relatives, are widely distributed throughout the Baltic Sea surface layer.

Our newly recovered AOA genomes enabled us to examine their biogeographical patterns by mapping metagenomes against them, along with GTDB references (Figure S7). We observed that *Ca*. N. balticolittoralis was predominant in surface waters of the western Baltic, whereas a closely related genome from a previously published Baltic Sea metagenome study (*Ca*. N. sp001437625 BACL13) (Hugerth et al. 2015) was more abundant in central and eastern surface waters, and again, *Ca*. N. balticoprofundus consistently dominated deep‐water samples. These mapping results largely reflect the patterns previously observed via phylotyping and rRNA gene sequencing, but with the new genomes established here, we were able to do so at a much more resolved whole‐genome level, exemplified by the finely resolved differences between the distributions of surface types *Ca*. N. balticolittoralis and BACL13.

### 3.5 Genome‐Based Trait Conservation and Variability of AOA

**FIGURE 6:**
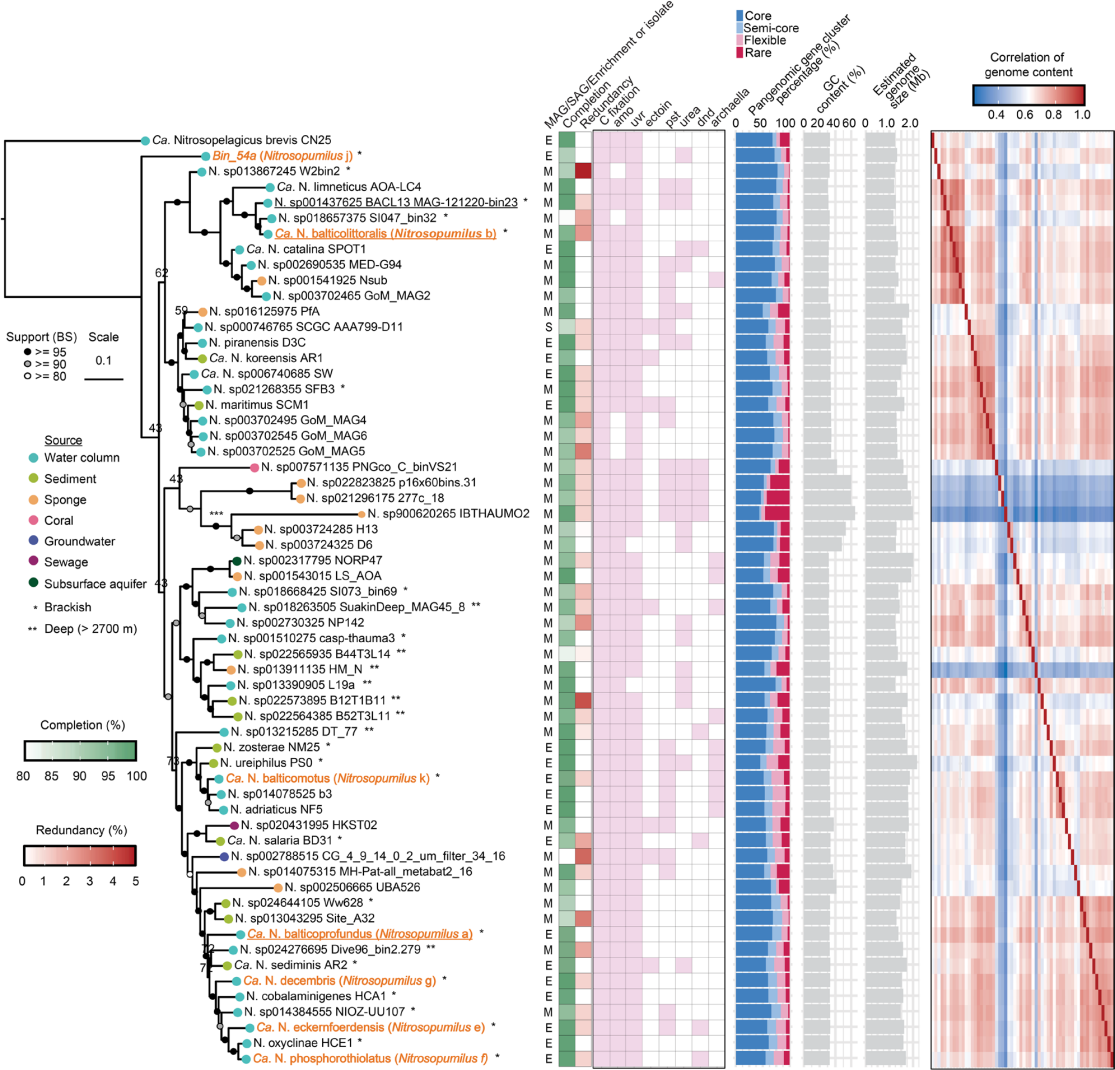
Phylogenomic and functional profiling of 59 *Nitrosopumilus* genomes. The phylogenomic tree was constructed based on archaeal single‐copy core genes, with *Ca*. Nitrosopelagicus brevis as an outgroup. Genomes recovered in this study are highlighted in orange, and three dominant *Nitrosopumilus* types from the Baltic Sea based on metagenomic mapping are underlined (see Figure S7). Genome sources are indicated by coloured dots at branch tips, and habitat types (brackish or deep sea > 2700 m) are marked with asterisks next to genome names. Basic genomic features (completion, redundancy, GC content, genome size and genome type [MAG/SAG/enrichment or isolate]) are displayed next to the tree. Selected functional gene sets are annotated in a presence/absence matrix (pink/white, respectively), where pink indicates the presence of > 50% of genes in each category: Carbon fixation (15 genes from 3HP/4HB cycle), ammonia oxidation (amo), UV repair (uvr), osmoregulation (ectoine), phosphate transport (pst), urease, DNA phosphorothioation (dnd) and motility (archaella). Pangenomic analysis shows the distribution of gene clusters across genomes, categorised as core, semi‐core, flexible, or rare, with their relative proportions shown for each genome. Genome content correlation was calculated based on gene presence/absence patterns across gene clusters. Asterisks (***) indicate branches that were artificially shortened by half for visualisation. Detailed information about individual genes is provided in Supplementary Data 5.

To contextualise Baltic Sea AOA within global *Nitrosopumilus* diversity, we constructed a phylogenomic tree incorporating genome metadata and functional gene annotations (Figure 6). Baltic Sea AOA were distributed across the tree, though they tended to be grouped with other brackish strains. *Ca*. N. balticolittoralis clustered closely with BACL13 and with *Ca*. *N. catalina* SPOT1, a surface‐associated strain from the northeast Pacific. By contrast, *Ca*. N. balticoprofundus grouped with a broader cluster of brackish‐origin genomes, including rare Baltic Sea strains such as *Ca*. N. phosphorothiolatus, *Ca*. N. eckernfoerdensis and *Ca*. N. decembris, all selectively enriched from the southwest Baltic Sea.

Next, in an effort to gain insight into the potential genetic drivers of niche differentiation of Baltic Sea AOA, we investigated the functional variability of their genomes. All genomes contained core genes essential for their lifestyle, including ammonia oxidation and carbon fixation, consistent with these core conserved features of the *Nitrosopumilus* genus. Additionally, while UV repair was found in almost all of the *Nitrosopumilus*, other auxiliary functions such as urease, motility, phosphorothioation, osmoregulation and phosphate transport were sparsely distributed with no clear phylogenetic pattern (Figure 6), similar to that observed recently (Rasmussen and Francis 2022). For example, while *Ca*. N. balticolittoralis lacks urease and phosphate binding proteins, its close relative BACL13 possesses both, suggesting fine‐scale metabolic divergence potentially linked to spatial distribution. Further support would, however, require environmental measurements such as urea concentrations.

**FIGURE 7:**
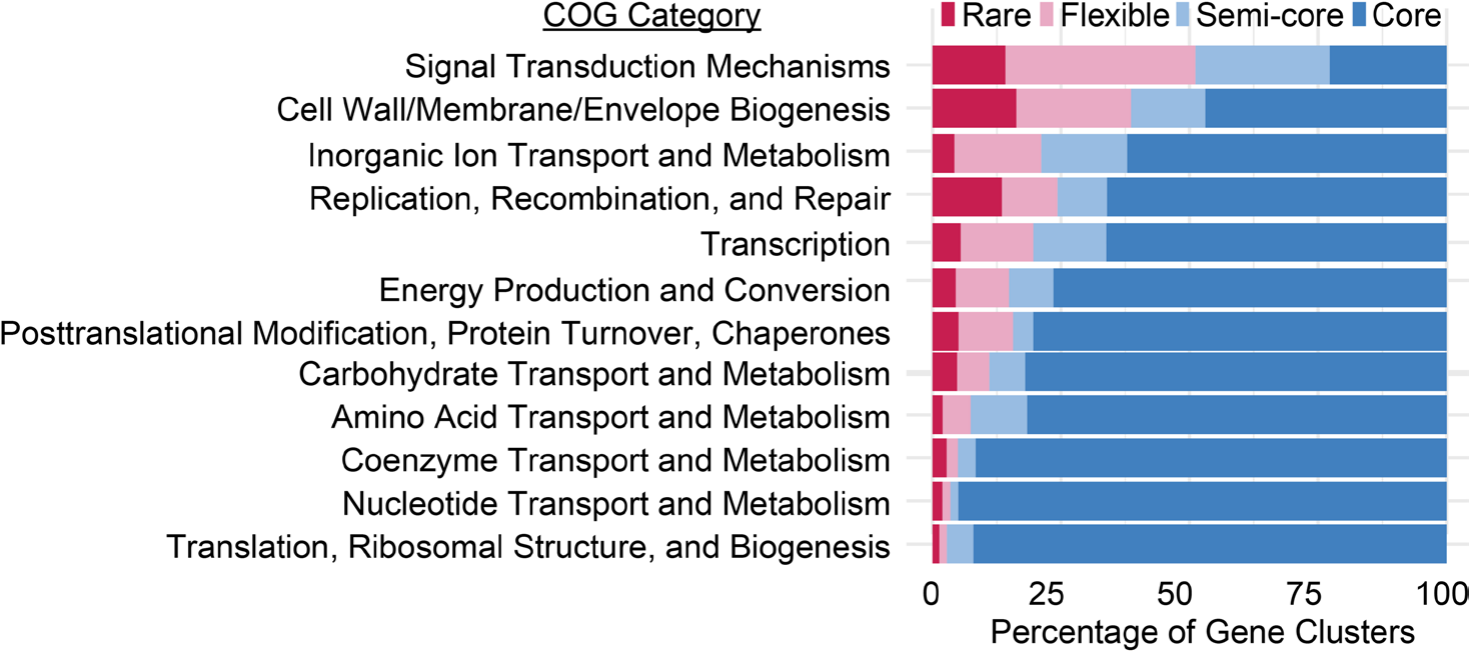
Distribution of COG20 functional categories across gene clusters in representative *Nitrosopumilus* genomes. COG20 categories are shown for genes classified as core, semi‐core, flexible and rare. Core and semi‐core genes are primarily associated with essential cellular functions, whereas flexible and rare genes show enrichment in environmental interaction processes, such as signal transduction, cell wall/membrane biogenesis and inorganic ion transport. Genes with unknown gene annotation, assigned to multiple categories, or from categories with fewer than 2000 genes were excluded from the analysis.

In addition to the targeted interrogation of these specific functions, we conducted a pangenomic analysis to examine the degree and patterns of gene variability across the *Nitrosopumilus* genus. Out of 14,118 gene clusters identified, only a small fraction was conserved across genomes, with the majority classified as flexible or rare (Figure S8). On average, core and semi‐core genes accounted for 75.3% ± 9.7% of each genome, whereas 13.3% ± 4.7% were flexible and 11.4% ± 9.7% were rare (Figure 6, rightmost panel). Although most flexible and rare genes lacked functional annotations (30.3% and 55%, respectively), the annotated subset was often involved in interacting with the external environment. For example, large proportions of genes associated with signal transduction, cell wall/membrane/envelope biogenesis and inorganic ion transport and metabolism were classified as semi‐core, flexible, or rare within the pangenome (Figure 7). The substantial proportion of flexible and unique genes highlights the extensive genomic diversity within *Nitrosopumilus*, but the implications remain to be fully evaluated as most of these genes remain unannotated via the conventional approaches employed.

## 4 Discussion

Our study provides an integrated analysis of the distribution and diversity of nitrifiers across the Baltic Sea, their high‐resolution temporal dynamics in the southwest Baltic Sea, and genomic insights into dominant nitrifiers, AOA. Our high‐resolution time series therefore provides the first fine‐scale temporal and seasonal view of nitrifier dynamics in Baltic surface waters, and the strain‐resolved profiles reveal ecological patterns that may reflect underlying niche differentiation within the nitrifier community. We observed clear temporal dynamics, particularly in the photic zone, where AOA and NOB abundances increased during late fall and winter. In addition, spatial patterns from metagenomic read recruitment to AOA genomes revealed regional variation in AOA strain composition across the southwest photic, central photic and aphotic waters. Comparative genome analysis of all dominant Baltic Sea AOA strains revealed a large degree of conservation overall, yet notable variability in key traits across genomes, providing a foundation for further mechanistic investigations into the environmental and functional drivers shaping nitrifier communities.

### 4.1 Diversity and Dynamics of Nitrifiers

Our observation revealed that nitrifiers were persistently common in the aphotic zones of the Baltic Sea, complementing rate measurements of ammonia oxidation in these areas (Enoksson 1986; Labrenz et al. 2010; Jäntti et al. 2018), as well as prior studies on AOA distribution within the Baltic Sea chemocline (Labrenz et al. 2010; Hietanen et al. 2012; Berg et al. 2014, 2015; Jäntti et al. 2018; Wittenborn et al. 2023). The vertical distribution is also similar to those observed especially in the brackish Black Sea, which has a similar permanent redoxcline to the Baltic Sea (Francis et al. 2005; Coolen et al. 2007; Lam et al. 2007). Similarly, nitrifiers, especially AOA, have been shown to be prevalent in the aphotic zone of the brackish Caspian Sea (Mehrshad et al. 2016; Miller et al. 2019), despite its weaker redoxcline, and have also been detected across large freshwater ecosystems (Klotz et al. 2022; Ngugi et al. 2023). In the Baltic and Black Sea, with their strong redoxclines, the apparent abundances of AOA throughout the aphotic zone contrast with measured nitrification rates, which are higher in the upper redoxcline (Enoksson 1986; Ward and Kilpatrick 1991; Lam et al. 2007; McCarthy et al. 2007; Jäntti et al. 2018). This pattern suggests either that per‐cell nitrification rates may be low in the depths not associated with the upper redoxcline, or that AOA persist for long periods of time until provided substrates, although more effort in this direction may be warranted.

Beyond the distributions in the aphotic zone, one of the most striking observations was in the photic zone, where we observed late‐fall and winter peaks in nitrifier abundance, especially among AOA and NOB. AOA have also been observed to have wintertime increases across various other marine surface waters, including the northern Atlantic Ocean (Haas et al. 2021), the Mediterranean Sea (Galand et al. 2010; Hugoni et al. 2013), the Arctic and Antarctic oceans (Williams et al. 2012; Wietz et al. 2021; Thiele et al. 2023), the Barents Sea (Thiele et al. 2023) and San Francisco Bay (Rasmussen and Francis 2022). However, such seasonality has not been clearly documented for the Baltic Sea, likely because earlier basin‐scale and time‐series studies used bacterial 16S rRNA primers that excluded AOA (Lindh et al. 2015; Latz et al. 2024).

During the late‐fall and winter, nitrifiers likely face reduced phototrophic competition from phytoplankton for nitrogen substrates under the low‐light winter conditions. In these periods, the strains dominating the surface communities may be better able to cope with light inhibition (Shears and Wood 1985; Merbt et al. 2012; Qin et al. 2014) in the case of AOA, for which mechanisms such as UV repair genes and diurnal cycling of activity have been reported (Hollibaugh et al. 2014; Pedneault et al. 2014).

Many different taxa correlated positively with nitrifiers in our time‐series, spanning eight distinct orders, whereas the sole negative correlation was between the dominant AOA and the summer‐abundant Burkholderiaceae RS62 marine group. Among the positively correlated ASVs were several lineages, such as SUP05, SAR324 and SAR406, that include relatives from deep‐sea habitats, some representatives of which, like nitrifiers, are chemoautotrophs (Sheik et al. 2014; Callbeck et al. 2021; Dede et al. 2022). We note, however, that these lineages show extensive diversity and are known, for example in the case of SAR324, to vary in their chemoautotrophy potential (Boeuf et al. 2021). Nevertheless, the co‐occurrence of these potential chemoautotrophic taxa in surface waters suggests that late‐fall and winter energy‐limited conditions may select for a shared set of ecological strategies that are akin to those typically associated with the aphotic zone. Future studies linking the observed diversity and seasonal dynamics of nitrifiers to direct measurements of nitrification and dark carbon fixation will be critical for understanding the ecological impacts of the observed dynamics.

Our data from Boknis Eck indicate that the nitrifier seasonality is not restricted to the Kiel Fjord, but is likely a broader phenomenon across the southwest Baltic Sea. At Boknis Eck, we also observed similar late‐fall and winter increases in the surface waters, along with a more persistent nitrifier population in the deeper depths (20 and 25 m). Although the long‐term interannual variation of nitrifiers is not yet known, the overall patterns we observed are consistent with long‐term biogeochemical data from Boknis Eck, which show clear fall and winter increases in nitrite and nitrate concentrations (Lennartz et al. 2014). At depth, the increased relative abundances and persistence may be due to the typically higher ammonia concentrations (Lennartz et al. 2014) as well as reduced light inhibition. Additionally, recent observations at Boknis Eck have reported elevated nitrous oxide concentrations throughout the water column from November to April (Ma et al. 2019), correlating with the timing of increased nitrifier populations in our study. The impact of surface nitrifier populations at both the Kiel Fjord and Boknis Eck during late‐fall and winter on carbon fixation and nitrification rates remains to be determined, alongside the factors that control these rates. Furthermore, these dynamics highlight the need to understand to what degree nitrifier populations are contributing to the production of nitrous oxide in the Baltic Sea, as in the global ocean (Santoro et al. 2011, 2021; Freing et al. 2012), compared with other processes like denitrification (Wan et al. 2023) and abiotic production (Leon‐Palmero et al. 2025).

### 4.2 Genomic Diversity of AOA

Our study, using both traditional and enrichment‐enabled metagenomics, successfully recovered all of the dominant AOA genomes observed in our dataset, as well as several rare AOA. Our results revealed that approximately 75% of the genes observed within individual *Nitrosopumilus* strains are core or semi‐core (i.e., commonly shared with other strains). The remaining flexible genes were often unannotated or involved in interactions with the external environment, such as cell membrane properties and ion transport. Such enrichment of flexible genes in poorly annotated functions and environmentally responsive traits has also been documented across diverse prokaryotes (Rodriguez‐Valera et al. 2009, 2016; Polz et al. 2013), including AOA (Suárez‐Moo et al. 2024). However, even with these genomes in hand, connecting the predicted functions of *Nitrosopumilus* overall to their distributions proved challenging. One reason for this is that our analyses demonstrated that even key ‘auxiliary’ functions, such as urea utilisation and motility, appear sporadically across genomes rather than being conserved within closely related lineages. This suggests that within *Nitrosopumilus*, these functions may be gained and lost via horizontal gene transfer (HGT), which complicates their association with particular lineages (Rasmussen and Francis 2022) and contributes to the variability in ecological functions even among closely related taxa. More broadly, the spatiotemporal distribution of *Nitrosopumilus* may reflect fine‐scale sequence variation or strain‐specific physiological traits that cannot be captured by gene‐content patterns alone (Jung et al. 2022; Suárez‐Moo et al. 2024).

One key difference we observed was the deep split between the dominant surface and deep types in our phylogenomic data, which suggests long‐term evolutionary divergence related to these respective environments. Our distributional data, coupled with the knowledge that the dominant surface types (*Ca*. N. balticolittoralis and BACL13) were closely related to a strain of AOA enriched from the surface Pacific Ocean (Ahlgren et al. 2017), thus, the taxa and their close relatives may be relatively more adapted to conditions in the photic zone. However, the degree to which these taxa are inhibited remains to be further explored, which is challenged by the difficulties in cultivating these organisms.

Going forward, efforts to characterise the distributions of *Nitrosopumilus* and their flexible genes across different ocean ecosystems and environments would provide valuable insights into AOA ecology. Such characterisations will help establish the mechanisms and key traits that lead to the success and niche variability of AOA in the ocean and will benefit from population genomics‐level analyses, such as the frequency of HGT to further understand *Nitrosopumilus* speciation processes (Polz et al. 2013). Additionally, complementary approaches like metatranscriptomics, metaproteomics and single‐cell activity measurements may help resolve the mechanisms and implications of their niche partitioning and seasonal photic zone dynamics.

## 5 Conclusions

By integrating high‐resolution time‐series and distributional data with genomic analysis, this study deepens understanding of the ecological niches and drivers shaping nitrifiers in the Baltic Sea. Consistent with other studies, we observed the persistent presence of nitrifiers beneath the photic zone. However, our results revealed that they are also prevalent from late fall to winter in the southwest Baltic Sea, tightly coupled to geochemistry in the water column, with AOA populations dominated by a single strain, based on ASV analysis and genome mapping. Additionally, a meta‐analysis combining our new metagenomic data with publicly available datasets revealed fine‐grained niche partitioning, with different strains being the most prevalent in the shallow western Baltic Sea compared to surface waters of the Baltic Sea proper and the aphotic zone depths of the Deep Baltic Sea. Our efforts to connect these distributions to genomic potential led to the enrichment of novel AOA strains and the recovery of all dominant AOA genomes from the Baltic Sea. These genomes showed that while the strains share most of their genes, each genome also contained large amounts of flexibility or novelty, particularly in genes linked to interactions with the external environment. How the genomic novelty and variation relates to the strong latitudinal, salinity and trophic gradients in the Baltic Sea remains to be more fully investigated, but the patterns suggest that the genomic variation may relate to both bottom‐up and top‐down controls. Moreover, understanding the intra‐annual and inter‐annual seasonality of the numerically dominant nitrifiers, AOA, will be key to understanding how these populations may respond to long‐term environmental changes, particularly given the low diversity of strains that dominate surface waters seasonally. Together, our time‐series and distributional study, coupled with the recovery of novel genomes, sets the stage for more detailed and mechanistic exploration into the bottom‐up and top‐down factors controlling the ecology and evolution of nitrifiers in the Baltic Sea and may ultimately extend these insights to other environments, including the deep ocean.

## Author Contributions

**Sukki Sookyoung Kim:** conceptualization, methodology, validation, writing – review and editing, writing – original draft, formal analysis, data curation. **Elisa D'Agostino:** methodology, validation, writing – review and editing, data curation. **David M. Needham:** conceptualization, writing – original draft, supervision, funding acquisition, writing – review and editing, resources, data curation.

## Funding

Helmholtz Young Investigator Grant and Deutsche Forschungsgemeinschaft Grant NE 2754/1‐1 to D.M.N.

## Conflicts of Interest

The authors declare no conflicts of interest.

## Supporting information


**Data S1:** emi70215‐sup‐0001‐supinfo_1.xlsx.

**Data S2:** emi70215‐sup‐0002‐supinfo_2.pdf.

## Data Availability

Previously unpublished environmental data utilised in the manuscript are available via Supplementary Data 1. Metagenome and amplicon short read datasets are available via NCBI BioProjects PRJNA1377132 and PRJNA1365321. The newly described AOA genome generated in this study have been deposited in NCBI under accessions GCA_054095705.1, GCA_054095935.1, GCA_054095825.1, GCA_054095695.1, GCA_054095835.1, GCA_054095715.1, and GCA_054095815.1. Alignments, phylogenetic trees, and amplicon sequencing data including ASV representative sequences, ASV tables, taxonomic assignments and metadata are available via Figshare (DOI: https://doi.org/10.6084/m9.figshare.c.7869515).

## Appendix A

### Protologues for New *Candidatus* Species Identified From Selective Enrichment and Metagenomics for six *Nitrosopumilus* Species

#### Description of *Candidatus* Nitrosopumilus balticoprofundus sp. nov.

*Candidatus* Nitrosopumilus balticoprofundus (bal.ti.co.pro.fun'dus. N.L. masc./fem. adj. balticus of or pertaining to the Baltic; L. adj. profundus deep; N.L. balticoprofundus predominance in deep Baltic Sea waters).

An archaeal species identified by selective enrichment‐enabled genomics. This genome originated from a Baltic Sea sample collected at Bornholm Basin, from a depth of 63 m, and was enriched using autoclaved Baltic Sea water amended with ammonium, phosphate, trace metals, and antibiotics. The enrichment was collected on a 0.2 μm filter, DNA extracted, sequenced by shotgun metagenomics with Illumina sequencing, assembled with SPAdes, and manually curated with Anvi'o. This species shows less than 95% average nucleotide identity to the type *Nitrosopumilus* genome (*N. maritimus* SCM1), and all representative genomes in the GTDB database. The phylogenetic position was analyzed using reference genomes from the GTDB database. The genome is estimated to be 97.09% complete and 0% contaminated. The GC content of the genome is 32.4%, and the genome size is 1.34 Mb with 40 contigs. The genome represents the numerically dominant archaeal taxon found in the deep Baltic Sea, according to mapping of metagenomes at 95% ANI. The type genome for this species is Bin_571‐3 which has been deposited at NCBI under accession GCA_054095705.1.

#### Description of *Candidatus* Nitrosopumilus eckernfoerdensis sp. nov.

*Candidatus* Nitrosopumilus eckernfoerdensis (ec.kern.foer.den'sis. N.L. masc./fem. adj. eckernfoerdensis referring to Eckernförde, a city in Germany near the location in the Baltic Sea from which the water for enrichment was collected).

An archaeal species identified by selective enrichment‐enabled genomics. This genome originated from a southwest Baltic Sea sample collected at Kiel Bight (Boknis Eck sampling location), from a depth of 5 m, and was enriched using autoclaved Baltic Sea water amended with ammonium, phosphate, trace metals and antibiotics. The enrichment was collected on a 0.2 μm filter, DNA extracted, sequenced by shotgun metagenomics with Illumina sequencing, assembled with SPAdes, and manually curated with Anvi'o. This species shows less than 95% average nucleotide identity to the type *Nitrosopumilus* genome (*N. maritimus* SCM1), and all representative genomes in the GTDB database. The phylogenetic position was analyzed using reference genomes from the GTDB database. The genome is estimated to be 99.03% complete and 0.97% contaminated. The GC content of the genome is 33.4% and the genome size is 1.62 Mb with 12 contigs. The genome represents a numerically rare archaeal taxon found in the southwestern Baltic Sea (Kiel Bight), according to mapping of metagenomes at 95% ANI and 16S rRNA sequence analysis. The type genome for this species is strain bin_32a which has been deposited at NCBI under accession GCA_054095935.1.

#### Description of *Candidatus* Nitrosopumilus balticomotus sp. nov.

*Candidatus* Nitrosopumilus balticomotus (bal.ti.co.mo tus. N.L. masc./fem. adj. balticus of or pertaining to the Baltic; L. adj. motus moved; N.L. balticomotus referring to the presence of an archaellum gene in its genome, a discerning feature of the genome).

An archaeal species identified by selective enrichment‐enabled genomics. This genome originated from a southwest Baltic Sea sample collected from the Kiel Bight (Kiel Fjord sampling location), from a depth of 1 m, and was enriched using filter‐sterilised (0.2 μm) Baltic Sea water amended with ammonium, phosphate, trace metals, and antibiotics. The enrichment was collected on a 0.2 μm filter, DNA extracted, sequenced by shotgun metagenomics with Illumina sequencing, assembled with SPAdes, and manually curated with Anvi'o. This species shows less than 95% average nucleotide identity to the type *Nitrosopumilus* genome (*N. maritimus* SCM1) and all representative genomes in the GTDB database. The phylogenetic position was analyzed using reference genomes from the GTDB database. The genome is estimated to be 97.09% complete and 0.97% contaminated. The GC content of the genome is 32.6%, and the genome size is 1.83 Mb with 36 contigs. The genome represents a numerically rare archaeal taxon found in the southwestern Baltic Sea (Kiel Bight), according to mapping of metagenomes at 95% ANI and 16S rRNA sequence analysis. The type genome for this species is strain bin_68KS which has been deposited at NCBI under accession GCA_054095825.1.

#### Description of Candidatus Nitrosopumilus decembris sp. nov.

*Candidatus* Nitrosopumilus decembris (de.cem'bris. L. adj. decembris pertaining to December; N.L. decembris indicating the month from which the water for enrichment was collected from Baltic Sea surface waters).

An archaeal species identified by selective enrichment‐enabled genomics. This genome originated from a southwest Baltic Sea sample collected from the Kiel Bight (Kiel Fjord sampling location), from a depth of 1 m, and was enriched using filter‐sterilised (0.2 μm) Baltic Sea water amended with ammonium, phosphate, trace metals, and antibiotics. The enrichment was collected on a 0.2 μm filter, DNA extracted, sequenced by shotgun metagenomics with Illumina sequencing, assembled with SPAdes, and manually curated with Anvi'o. This species shows less than 95% average nucleotide identity to the type *Nitrosopumilus* genome (*N. maritimus* SCM1) and all representative genomes in the GTDB database. The phylogenetic position was analyzed using reference genomes from the GTDB database. The genome is estimated to be 100% complete and 0.97% contaminated. The GC content of the genome is 33.3%, and the genome size is 1.5 Mb with 11 contigs. The genome represents the numerically rare archaeal taxon found in the southwestern Baltic Sea (Kiel Bight), according to mapping of metagenomes at 95% ANI and 16S rRNA sequence analysis. The type genome for this species is strain bin_6a which has been deposited at NCBI under accession GCA_054095695.1.

#### Description of *Candidatus* Nitrosopumilus phosphorothiolatus sp. nov.

*Candidatus* Nitrosopumilus phosphorothiolatus (phos.pho.ro.thi.o.la tus. N.L. neut. n. phosphorus relating to phosphate; G. n. thiol relating to sulphur compounds; N.L. phosphorothiolatus referring to the presence of a phosphothioation pathway in its genome, a discerning feature of the genome).

An archaeal species identified by selective enrichment‐enabled genomics. This genome originated from a southwest Baltic Sea sample collected from the Kiel Bight (Kiel Fjord sampling location), from a depth of 1 m, and was enriched using filter‐sterilised (0.2 μm) Baltic Sea water amended with ammonium, phosphate, trace metals, and antibiotics. The enrichment was collected on a 0.2 μm filter, DNA extracted, sequenced by shotgun metagenomics with Illumina sequencing, assembled with SPAdes, and manually curated with Anvi'o. This species shows less than 95% average nucleotide identity to the type *Nitrosopumilus* genome (*N. maritimus* SCM1) and all representative genomes in the GTDB database. The phylogenetic position was analyzed using reference genomes from the GTDB database. The genome is estimated to be 99.51% complete and 0.97% contaminated. The GC content of the genome is 32.8%, and the genome size is 1.7 Mb with 11 contigs. The genome represents the numerically rare archaeal taxon found in the southwestern Baltic Sea (Kiel Bight), according to mapping of metagenomes at 95% ANI and 16S rRNA sequence analysis. The type genome for this species is strain bin_7KS which has been deposited at NCBI under accession GCA_054095835.1.

#### Description of *Candidatus* Nitrosopumilus balticolittoralis sp. nov.

*Candidatus* Nitrosopumilus balticolittoralis (bal.ti.co.lit.to.ra.lis. N.L. masc./fem. adj. balticolittoralis, from Balticum (the Baltic Sea) and littoralis (pertaining to the shore or littoral waters), referring to its occurrence in the coastal surface waters of the Baltic Sea).

An archaeal species identified by metagenomics. This genome originated from a southwest Baltic Sea sample collected from the Kiel Bight at Boknis Eck sampling location from a depth of 25 m in February 2022. The seawater was collected on a 0.2 μm filter, DNA extracted, sequenced by shotgun metagenomics with Illumina sequencing, assembled with SPAdes, and manually curated with Anvi'o. This species shows 98.5% average nucleotide identity to the closest representative genome in the GTDB database (sp014384555). This closest relative is also a strain identified by metagenomics from the Black Sea, which has not been formally described, and has only a species place‐holding number (sp014384555). The phylogenetic position was analyzed using reference genomes from the GTDB database. The genome is estimated to be 96.12% complete and 2.27% contaminated. The GC content of the genome is 32.5%, and the genome size is 1.26 Mb with 50 contigs. The genome represents the numerically dominant archaeal taxon found in the southwestern Baltic Sea (Kiel Bight), according to mapping of metagenomes at 95% ANI. The type genome for this species is strain metabat.KBP569_Feb_25m_nospike.7 which has been deposited at NCBI under acccession GCA_054095715.1.
