# Supplementary material for: Dynamics and Eco‐Genomics of Baltic Sea Nitrifiers: Seasonality, Niches, Interactions and Genomic Uniqueness
Source: Environ Microbiol. 2026 Jan 7;28(1):e70215. doi: 10.1111/1462-2920.70215 (PMC12780485; doi:10.1111/1462-2920.70215)
Supplement: Supplementary file 2 — Data S2: emi70215‐sup‐0002‐supinfo_2.pdf. [file EMI-28-e70215-s002.pdf]

Supplementary Figures and Figure Legends

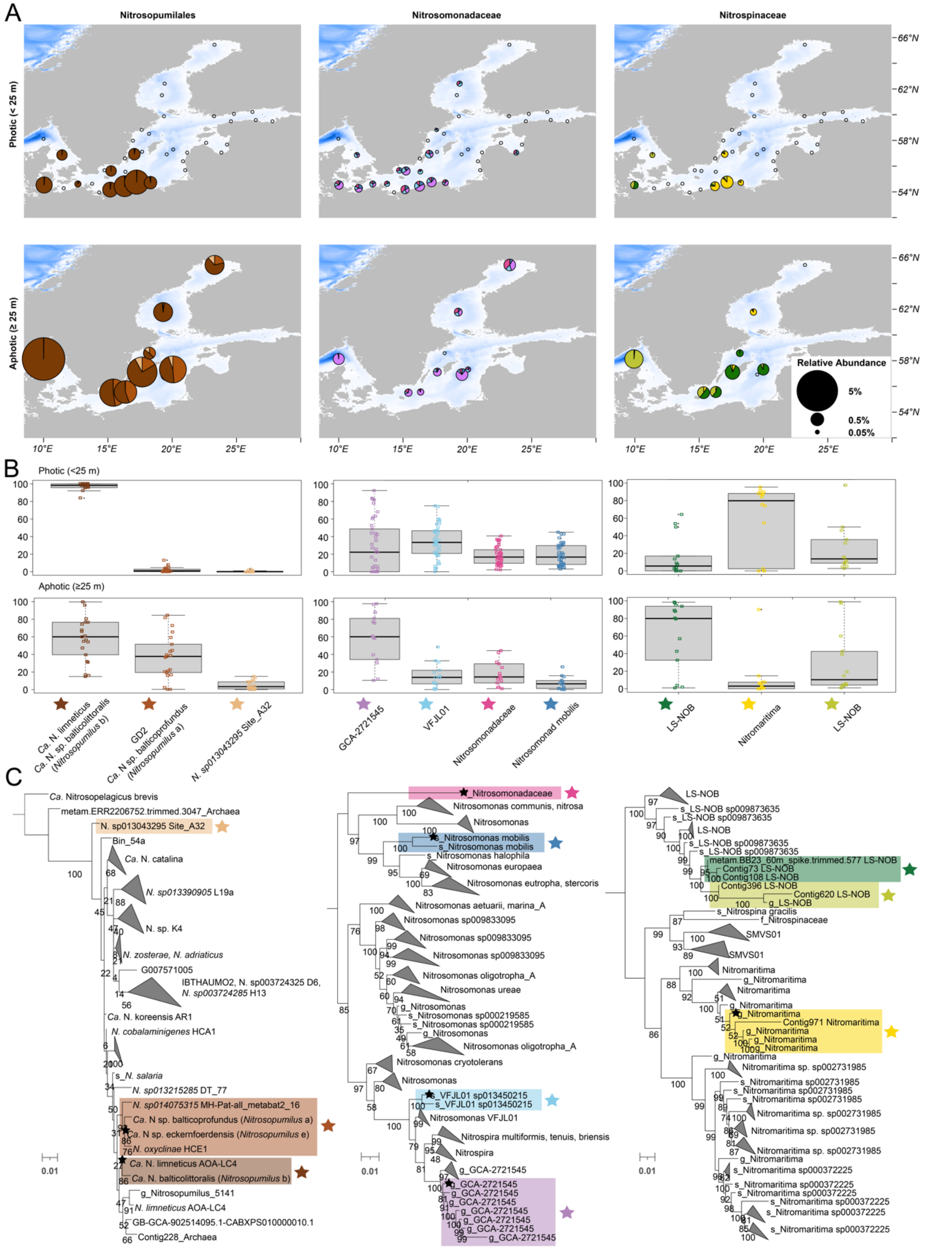

S Fig 1: Distributions of nitrifiers and phylotypes across the Baltic Sea. The figure is based on the same data as Fig. 1, but includes a breakdown of individual phylotypes

observed based on phylogenetic placement. As in Fig. 1, A) The pie size indicates the average relative abundance of each family at a given location (samples within 1° of latitude and longitude are averaged) with colors representing the respective phylotypes. The upper panels show surface samples (0-25 m), and the lower panels show deep samples (≥ 25 m). Open black circles indicate no detection. Background shading (white and blue) represents water column depth. B) Box-and-whisker plots summarizing the proportion of each phylotype across all samples within each depth range. C) The phylogenetic trees used for phylotype assignment are shown. Collapsed branches represent phylotypes not detected or present only at negligible levels in the dataset. Details on phylotype assignment are provided in the Methods.

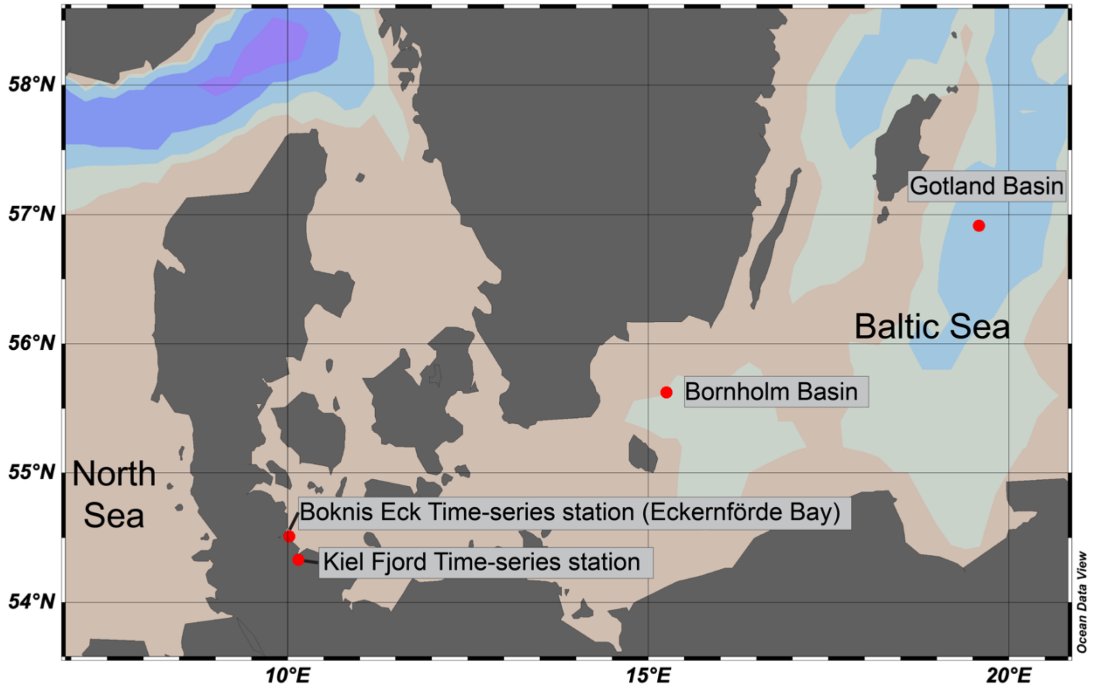

S. Fig 2: Map of sampling locations included in this study. Sampling sites include: the Kiel Fjord Time-series Station (sampled twice-weekly from October 2021 to May 2023); the Boknis Eck Time-series Station in Eckernförde Bay (sampled monthly from January 2022 to June 2023); and broader Baltic Sea sampling sites at Bornholm Basin and Gotland Basin (sampled in September 2022). Locations of previously published metagenomic datasets are not shown on this map.

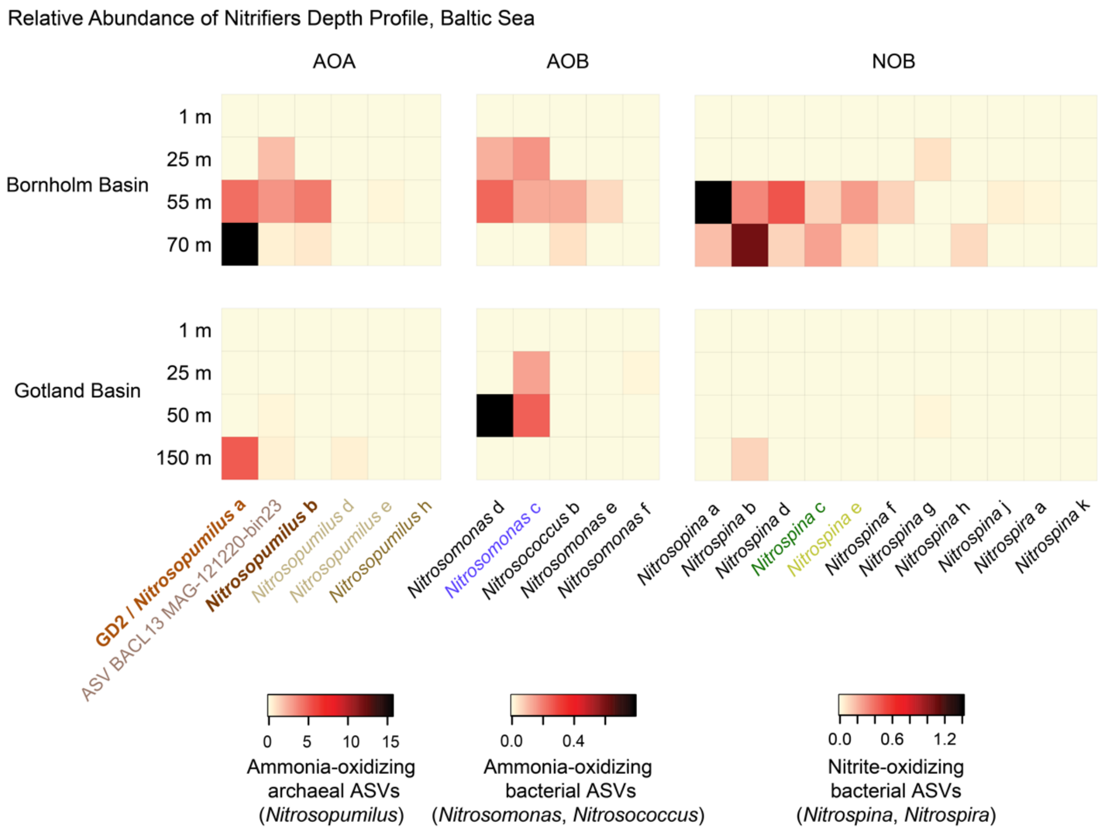

S. Fig 3: Depth distributions of nitrifiers at two Baltic Sea locations (Bornholm and Gotland Basins). Sampling conducted in early September revealed that nitrifier communities were barely detected in surface waters but maintained substantial populations in aphotic waters (≥ 25 m). AOA dominated the nitrifier communities, particularly Nitrosopumilus a, which showed the highest relative abundance in deep waters at both sites. While AOA and NOB maintained substantial populations in deeper waters, AOB abundance was limited to intermediate depths. These patterns suggest differential adaptation of nitrifiers under oxygen-limited conditions, with Nitrosopumilus a and Nitrosopina b persisting in deeper waters while AOB were mostly restricted to intermediate depths. ASV colors are matched to those used in Fig. 2.

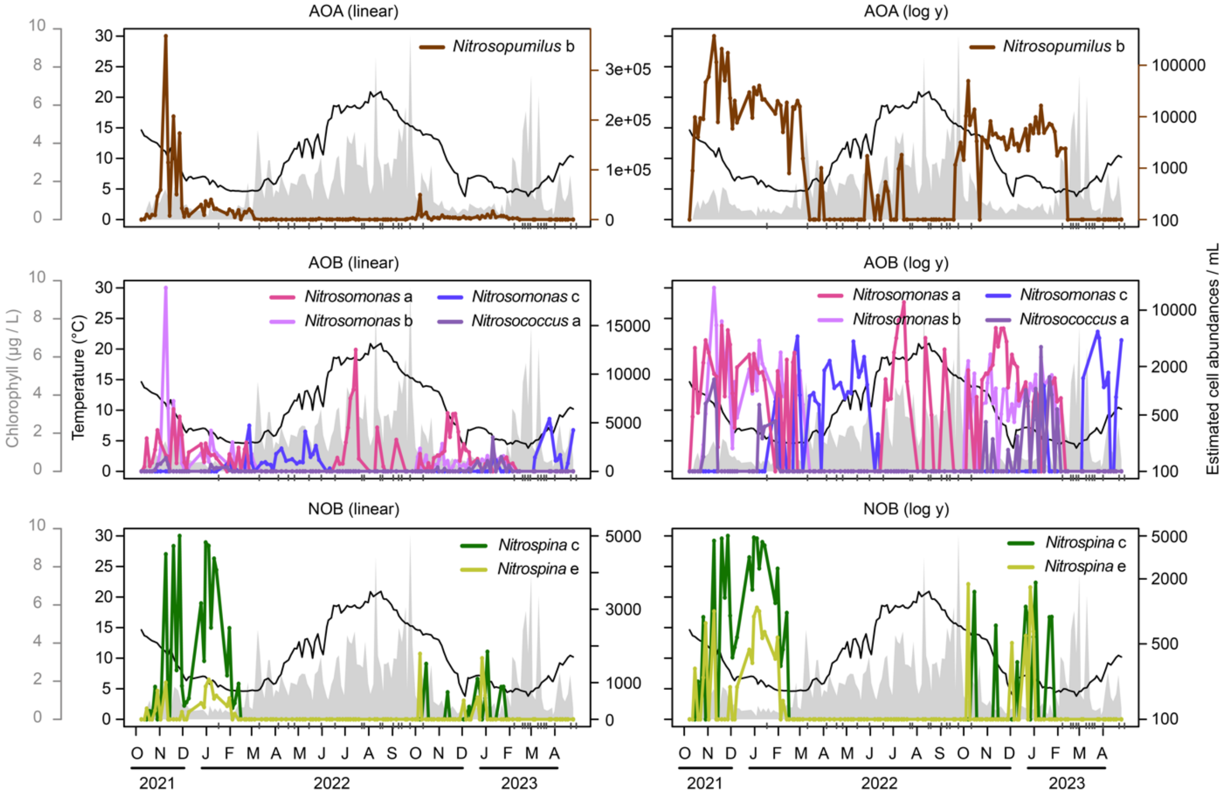

S. Fig. 4: Time-series dynamics of nitrifier ASVs at the Kiel Fjord Time-series Station based on estimated cell abundances. These values are based on normalization with spike-in of exogenous bacterial cells (see Methods). The left side panels show values on a linear scale, while the right side panels use a logarithmic scale (estimated abundance +100 to accommodate zeros and missing (NA) values for visualization). Time points for which the spike-in was not detected are indicated by ticks along the x-axis. Spearman correlations between estimated cell abundances and relative abundances are provided in Supplementary Data 3.

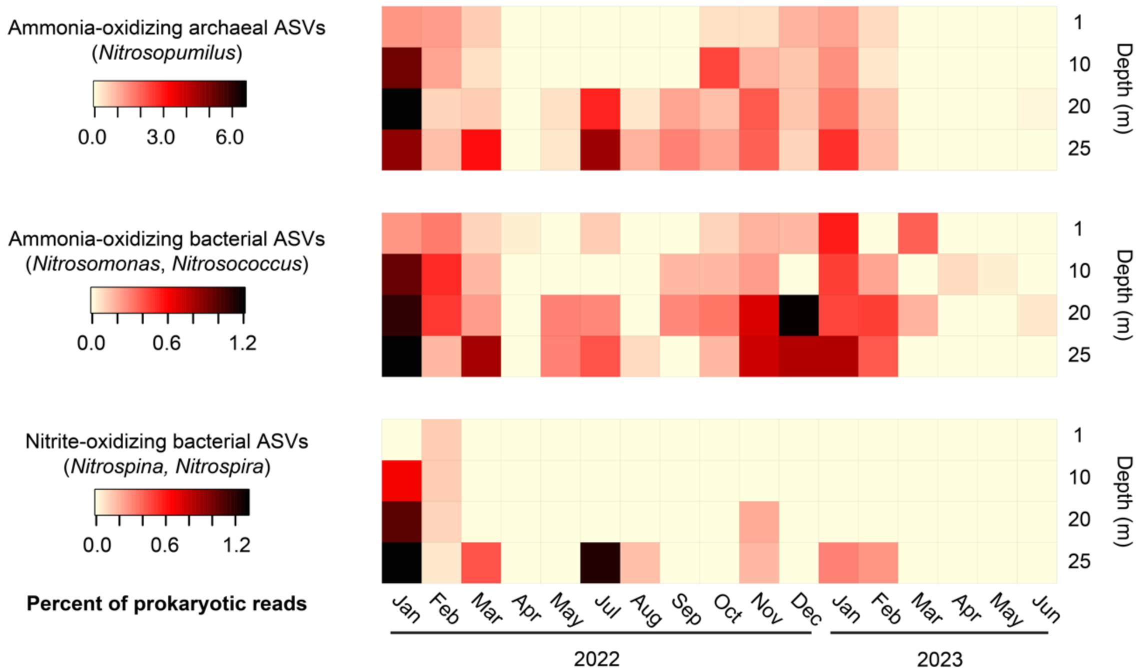

S. Fig 5: Depth and temporal dynamics of nitrifiers at Boknis Eck Time-series Station in the southwest Baltic Sea. In order to contextualize the surface dynamics observed in the Kiel Fjord Station, we examined nitrifier dynamics from 1 m to 25 m at the Boknis Eck Time-series Station over the same period. This monthly depth-integrated time-series demonstrated nitrifiers were also abundant throughout the water column during the winter, but were even more abundant at deeper depths (20 m and 25 m). During summer, nitrifier populations persisted in the deeper waters (mainly 20 and 25 m), occasionally showing increased abundance.

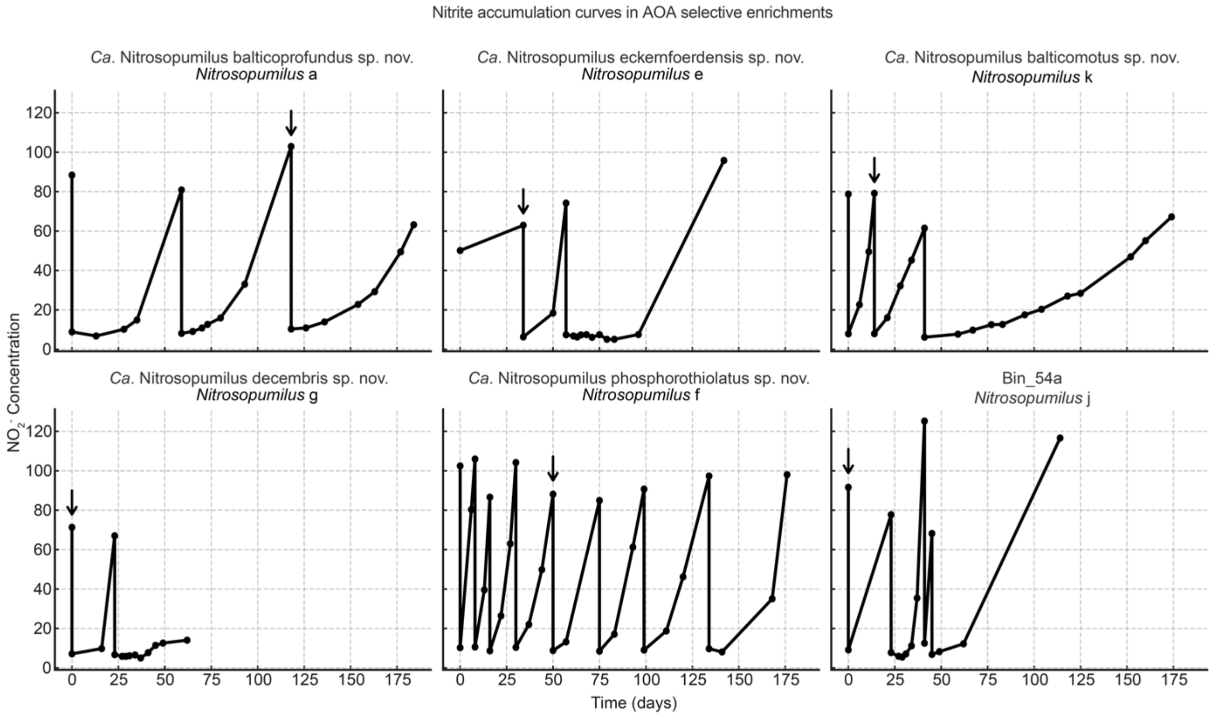

S. Fig. 6: Nitrite accumulation and genome sampling points of AOA-selective enrichment cultures. To obtain genome sequences of multiple AOA from the Baltic Sea, aged, filtered, and/or autoclaved Baltic Sea water was amended with ammonium, antibiotics, and inorganic nutrients, then inoculated (10% vol: vol) with Baltic Sea water from various sources (Supplementary Data 4) and incubated in the dark. After weeks to months of incubation, enrichments exhibiting substantial increases in nitrite were transferred into fresh media. Following this, the cultures were monitored and transferred more frequently to further enrich AOA. Arrows indicate the time points when incubations were sampled for amplicon and genome sequencing. In some cases, such as *Ca.* N. balticoprofundus, repeated growth occurred over several months, whereas in others, such as *Ca.* N. decembris, the growth could not be maintained over more than two months after first detection.

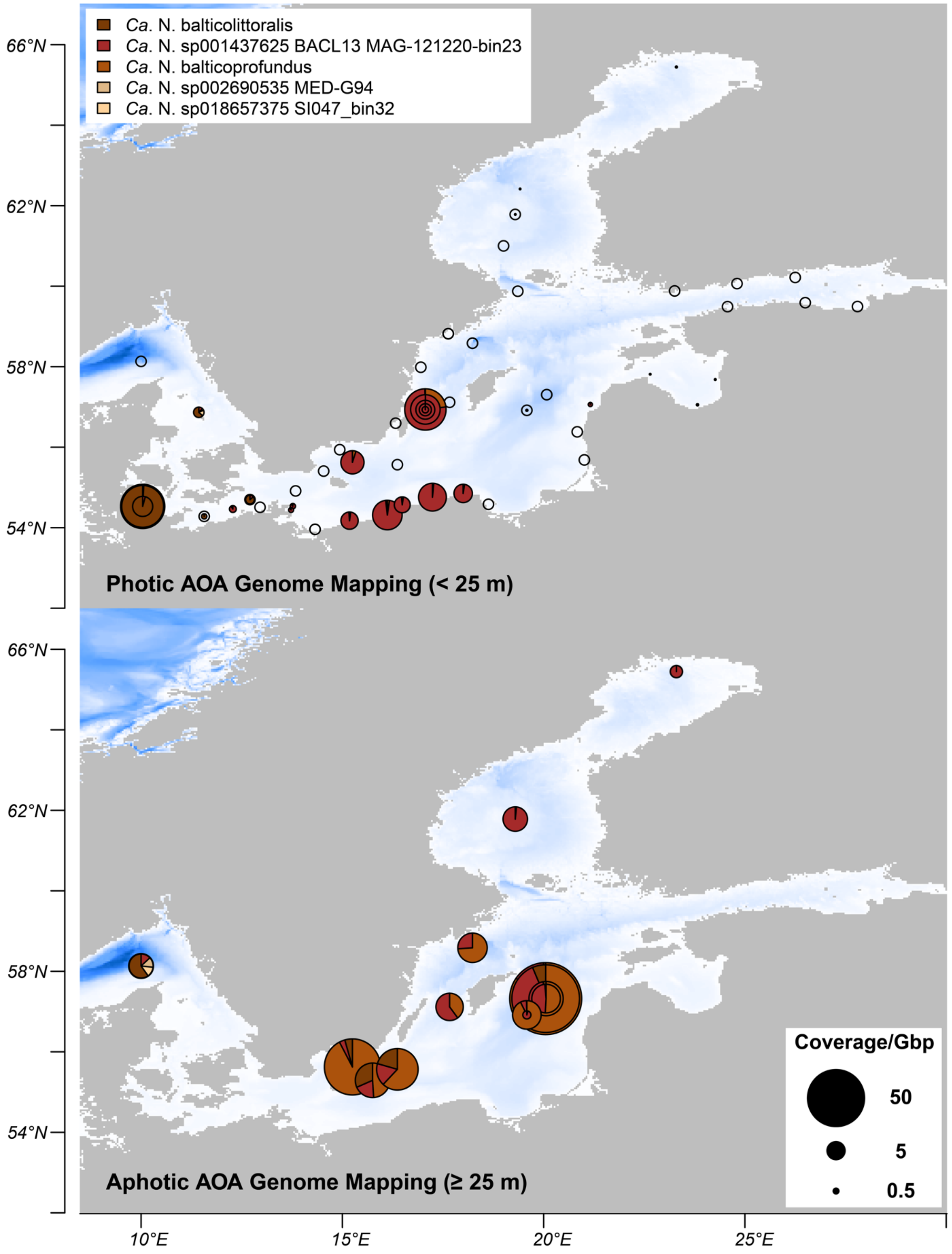

S. Fig. 7. Coverage of AOA genomes across the Baltic Sea based on metagenomic read recruitment. Metagenomic reads from Baltic Sea samples were mapped against 59

representative *Nitrosopumilus* genomes and *Nitrosopelagicus* as an outgroup, including both newly recovered and GTDB reference genomes (see Methods). Only genomes with cumulative coverage > 25x across all samples are shown. It reveals three dominant *Nitrosopumilus* types with distinct spatial distributions: *Ca.* N. kielensis dominated surface waters in the western Baltic Sea, *Ca.* N. sp001437625 BACL13 in the central and eastern basins, and *Ca.* N. balticoprofundus in deep waters.

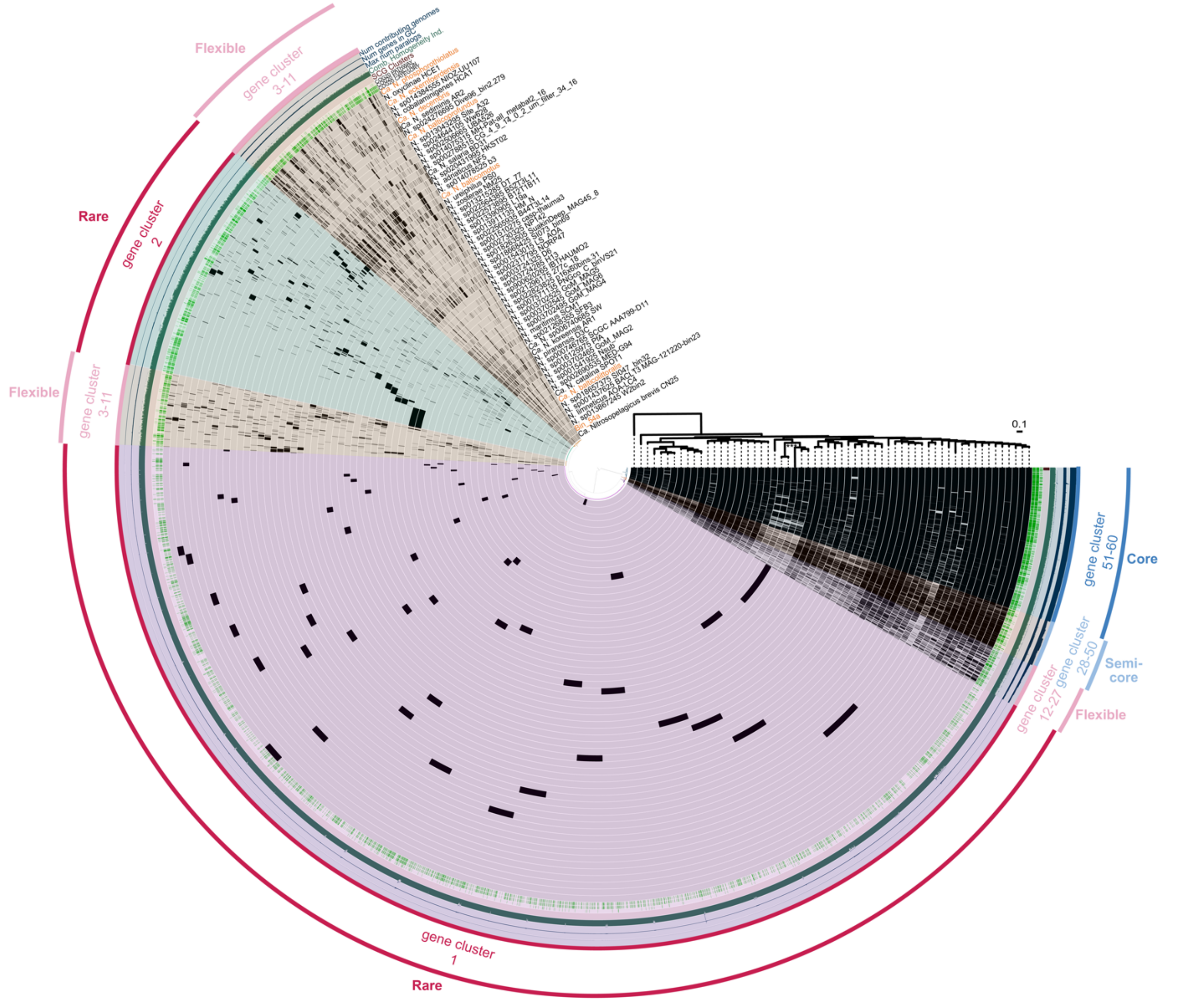

S. Fig 8: Pangenome analysis of 60 representative AOA genomes. Each bar in the circularized plot represents a gene cluster, filled if a given genome contributes a gene to that cluster. The outermost layer categorizes gene clusters as core (present in 51–60 genomes), semi-core (28–50), flexible (3–27), or rare (1-2). In total, 14,118 gene clusters were identified, of which 7.84% were core, 2.34% semi-core, 14.15% flexible, and 75.67% rare. The inner dendrogram clusters homologous gene families based on their presence/absence patterns across genomes. The genome order follows the phylogenomic tree shown in Fig. 6, which is also displayed in the middle-right of this figure. Genomes marked with *** indicate deep-branched, sponge-associated genomes. The outer layers indicate information for each gene cluster: 1. number of contributing genomes, 2. number of genes in the cluster, 3. maximum number of paralogs, 4. combined homogeneity index (indicative of similarity of sequences within a gene cluster), 5. SCG Clusters; single copy gene clusters, 6. COG20 PATHWAY, 7. COG20 FUNCTION, 8. COG20 CATEGORY. That is, points 6-8 indicate where a gene has an annotation in the COG database. Most of the rare genes lack COG annotations, while SCGs typically have annotations.
